# Convergent Evolution of Hydrogenosomes from Mitochondria by Gene Transfer and Loss

**DOI:** 10.1093/molbev/msz239

**Published:** 2019-10-24

**Authors:** William H Lewis, Anders E Lind, Kacper M Sendra, Henning Onsbring, Tom A Williams, Genoveva F Esteban, Robert P Hirt, Thijs J G Ettema, T Martin Embley

**Affiliations:** 1 Institute for Cell and Molecular Biosciences, Newcastle University, Newcastle-Upon-Tyne, United Kingdom; 2 Department of Cell and Molecular Biology, Uppsala University, Uppsala, Sweden; 3 Laboratory of Microbiology, Department of Agrotechnology and Food Sciences, Wageningen University, Wageningen, The Netherlands; 4 School of Biological Sciences, University of Bristol, Bristol, United Kingdom; 5 Department of Life and Environmental Sciences, Bournemouth University, Poole, United Kingdom

**Keywords:** evolution, genomics, hydrogenosomes, mitochondria, microbial eukaryotes, anaerobic metabolism

## Abstract

Hydrogenosomes are H_2_-producing mitochondrial homologs found in some anaerobic microbial eukaryotes that provide a rare intracellular niche for H_2_-utilizing endosymbiotic archaea. Among ciliates, anaerobic and aerobic lineages are interspersed, demonstrating that the switch to an anaerobic lifestyle with hydrogenosomes has occurred repeatedly and independently. To investigate the molecular details of this transition, we generated genomic and transcriptomic data sets from anaerobic ciliates representing three distinct lineages. Our data demonstrate that hydrogenosomes have evolved from ancestral mitochondria in each case and reveal different degrees of independent mitochondrial genome and proteome reductive evolution, including the first example of complete mitochondrial genome loss in ciliates. Intriguingly, the FeFe-hydrogenase used for generating H_2_ has a unique domain structure among eukaryotes and appears to have been present, potentially through a single lateral gene transfer from an unknown donor, in the common aerobic ancestor of all three lineages. The early acquisition and retention of FeFe-hydrogenase helps to explain the facility whereby mitochondrial function can be so radically modified within this diverse and ecologically important group of microbial eukaryotes.

## Introduction

Mitochondria are an ancestral feature of eukaryotic cells that have diversified in form and function during their separate evolution in eukaryotes under different living conditions, producing a spectrum of homologous organelles with different proteomes and phenotypes (Embley and Martin 2006; Muller et al. 2012; Stairs et al. 2015). Among the most interesting of these mitochondrial homologs are the hydrogenosomes (Müller 1993) found in anaerobic free-living and parasitic microbial eukaryotes. Hydrogenosomes produce H_2_ using the enzyme FeFe-hydrogenase, a type of metabolism that has been typically associated with bacteria rather than eukaryotes (Müller 1993; Embley et al. 1997; Horner et al. 2000; Muller et al. 2012; Stairs et al. 2015). The evolution of hydrogenosomes and the origins of their anaerobic metabolism are actively debated (Martin and Müller 1998; Martin et al. 2015; Stairs et al. 2015; Spang et al. 2019). Here, we have addressed these questions by investigating the repeated convergent evolution of hydrogenosomes from mitochondria among free-living anaerobic ciliates.

Ciliates provide an excellent system for studying the evolutionary transition from mitochondria to hydrogenosomes because anaerobic, hydrogenosome-containing ciliates are interleaved among aerobic, mitochondria-bearing forms in the ciliate tree (Embley et al. 1995; Fenchel and Finlay 1995). Previous work has also shown that the hydrogenosomes of the anaerobic ciliate *Nyctotherus ovalis* have a mitochondrial genome, providing direct molecular evidence of their mitochondrial ancestry (Akhmanova et al. 1998; Boxma et al. 2005; de Graaf et al. 2011). The retention of a mitochondrial genome contrasts with better-studied hydrogenosome-containing protists like *Trichomonas*, where the organellar genome has been entirely lost, and where close metamonad relatives lack classical aerobic mitochondria for comparison (Stairs et al. 2015; Leger et al. 2017). Anaerobic ciliates thus provide a rare opportunity to investigate the plasticity of mitochondrial function and the repeated convergent evolution of hydrogenosomes within a phylogenetically coherent and diverse lineage (Embley et al. 1995, 1997; Akhmanova et al. 1998; Boxma et al. 2005; de Graaf et al. 2011).

Anaerobic ciliates with hydrogenosomes are unusual among eukaryotes because they typically harbor endosymbiotic archaea, which in some cases form intricate physical interactions with ciliate hydrogenosomes (van Bruggen et al. 1983; Finlay and Fenchel 1991; Embley et al. 1992; Fenchel and Finlay 1995). The endosymbionts are methanogens that use the H_2_ produced by hydrogenosomes as an electron donor for ATP-producing methanogenesis (Fenchel and Finlay 1995). Physiological studies (Fenchel and Finlay 1991, 1995) have suggested that the endosymbionts provide an electron sink that can compensate for the reduced oxidative capacity of ciliate hydrogenosomes. Consistent with this, published data (Fenchel and Finlay 1991) have demonstrated that large ciliates like *Metopus contortus* and *Plagiopyla frontata* grow better when they harbor methanogens. However, there are currently no published data describing the proteomes and electron transport chains (ETCs) of the hydrogenosomes of either species to identify the molecular details underpinning their symbioses.

In the present study, we have investigated the repeated convergent evolution of ciliate hydrogenosomes (Embley et al. 1995), in representative species from three taxonomically distinct anaerobic lineages. Small-scale genomic amplification after hydrogenosome enrichment was used to recover organellar genome sequences for individual species, and these data were complemented by nuclear and organellar transcriptomics data generated by single-cell RNAseq. The molecular data sets generated were used to reconstruct hydrogenosome metabolism for *Cyclidium porcatum*, *M. contortus* and *P. frontata*, and phylogenetics was used to investigate the evolutionary history and origins of the key anaerobic enzymes for H_2_ generation.

## Results and Discussion

### Phylogenetic Analysis Supports the Independent Origins of Hydrogenosomes in Different Anaerobic Ciliate Lineages

**Fig. 1.**
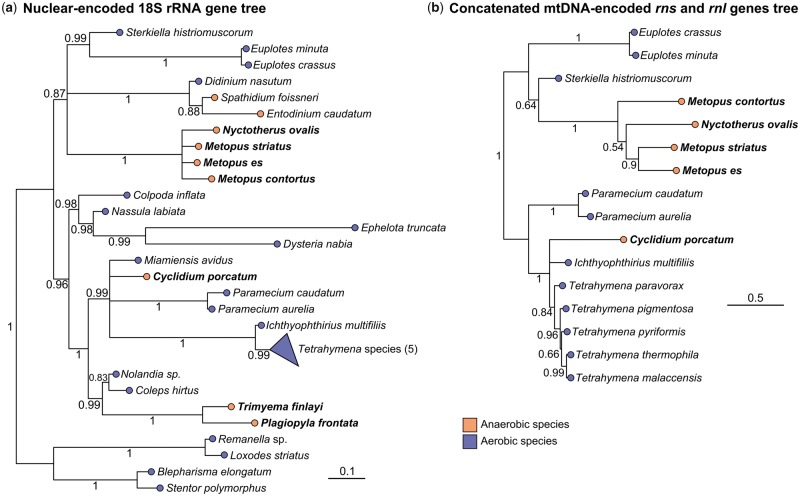
Ciliate rRNA gene phylogenies inferred using the program Phylobayes MPI from alignments of nuclear-encoded 18S rRNA genes (*a*), and from concatenated alignments of their mtDNA-encoded *rns* and *rnl* genes (*b*), using the CAT + GTR model. Both trees include aerobic and anaerobic representative species. The species investigated in the present study are highlighted in bold text, support values represent posterior probabilities, and scale bars represent the number of substitutions per site.

Selective enrichment culturing was used to isolate *M. contortus* and *P. frontata* from marine sediments and *C. porcatum*, *Metopus es*, *Metopus striatus*, and *Trimyema finlayi* (Lewis et al. 2018) from freshwater sediments. Cells of *N. ovalis* were isolated directly from the digestive tract of cockroaches. Phylogenetic analyses of 18S rRNA sequences from the isolates using the best-fitting CAT-GTR model (Lartillot et al. 2013) confirmed previous analyses using simpler models (Embley et al. 1995) showing that these species represent three distinct hydrogenosome-containing lineages: Armophorea (*Metopus* and *Nyctotherus*), Plagiopylea (*Plagiopyla* and *Trimyema*), and *C. porcatum*, nested among aerobic ciliates (fig. 1*a*). Our broad taxonomic sampling thus provides an opportunity to compare and contrast the molecular details of three separate events of hydrogenosome evolution.

### Some of the Sampled Anaerobic Ciliates Retain a Mitochondrial Genome

**Fig. 2.**
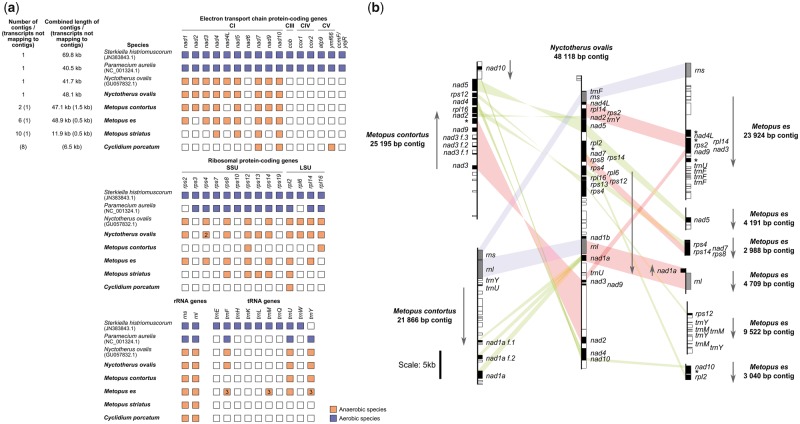
(*a*) A table showing the genes of known function predicted from the mtDNA of ciliates sequenced in the present study (species names in bold) and three that were sequenced by previous studies (Pritchard et al. 1990; de Graaf et al. 2011; Swart et al. 2012). Filled/colored boxes indicate genes that are encoded by the mtDNA for each species and empty/white boxes indicate genes that were not identified but are present in the mtDNA of other ciliates. In cases where multiple copies of a particular gene were found, encoded by the mtDNA of one species, the number of copies is indicated in the corresponding box for that gene. The mitochondrial complexes to which the products of the genes listed belong are indicated using the abbreviations CI, CIII, CIV, and CV, which correspond to the electron transport chain complexes I, III, IV, and V (F_0_F_1_ ATP-synthase), respectively. SSU and LSU correspond to the small and large mitochondrial/hydrogenosomal ribosome subunits, respectively. (*b*) A genomic map of gene positions for mtDNA contigs sequenced from *Metopus contortus*, *Nyctotherus ovalis*, and *Metopus es*, in the present study. Predicted protein-coding genes with homologs in other eukaryotes are represented by black boxes, predicted protein-coding genes with no homologs in any other organisms are represented by white boxes, and predicted RNA genes are represented by gray boxes. Predicted protein-coding genes that have detectable homologs from mtDNA of other ciliates, but from no other organisms outside ciliates, are labeled (*). Sections of colinear gene order between two mtDNA from different species are indicated with red bands, protein-coding genes with no discernible colinearity between mtDNA from two species are indicated with green bands, and the relative positions of rRNA genes are indicated with blue bands. Arrows indicate the direction of transcription. Fragment copy genes present in *Metopus contortus* mtDNA sequence are labeled (*f.*). Genes listed in the table (*a*) that are not shown in a corresponding genomic map for the same species (*b*) were only detected from transcript data that did not form part of the genomic contig assemblies. The DNA sequences corresponding to this figure are available in supplementary data 2, Supplementary Material online.

To investigate whether the hydrogenosomes of the sampled anaerobic ciliates have retained a mitochondrial genome (mtDNA), we generated new molecular data sets for *M. contortus*, *M. es*, *M. striatus*, *P. frontata*, *T. finlayi* (Lewis et al. 2018), and *C. porcatum* using multiple displacement amplification and genomic sequencing of DNA from hydrogenosome-enriched samples, and complemented these data using single-cell RNAseq (supplementary table 1, Supplementary Material online). We also produced new data for *N. ovalis* to complement the partial mtDNA sequence already available for this species (Akhmanova et al. 1998; de Graaf et al. 2011). We found evidence for mtDNA in samples from *M. contortus*, *M. es*, *M. striatus*, and *N. ovalis*, including ribosomal RNA genes that cluster strongly with mitochondrial sequences from related aerobic mitochondria-containing ciliates (fig. 1*b*). These data suggest that retention of mtDNA may be a conserved feature of the exclusively anaerobic class Armophorea (Fenchel and Finlay 1995; Lynn 2008) that contains *Metopus* and *Nyctotherus*. We did not detect any mtDNA in the samples for *C. porcatum*, but we did detect transcripts in the *C. porcatum* RNAseq data for mitochondrial protein-coding genes (fig. 2) and for mitochondrial LSU and SSU rRNA (fig. 1*b*), which suggests that *C. porcatum* has also retained a mitochondrial genome. By contrast, we found no molecular evidence for mtDNA in the hydrogenosomes of *P. frontata* or *T. finlayi*. This suggests that species in this clade have, so far uniquely among ciliates, completely lost the mitochondrial genome during hypoxia-driven reductive evolution of their hydrogenosomes.

We assembled a single 48,118-bp contig of mtDNA for *N. ovalis* (fig. 2*a* and *b*) which is similar to the size (∼48 kb) of its mtDNA as previously estimated from Southern blots (de Graaf et al. 2011). Based on the size of the mtDNA of *N. ovalis*, we estimate that we also obtained near-complete mtDNA data for *M. contortus* (48,599 bp) and *M. es* (48,877 bp), assembled in several short contigs and transcripts (fig. 2). One end of the new contig from *N. ovalis* was found to contain a 38-bp sequence (TATTGTAATACTAATAATATGTGTGTTAATGCGCGTAC) that is repeated in tandem three times, resembling the structure of telomeres from the mtDNA of other ciliates (Morin and Cech 1986). This suggests that the mtDNA of this species is a single linear chromosome similar to the mtDNA of several aerobic ciliates (Pritchard et al. 1990; Burger et al. 2000). The gene content of the new *N. ovalis* mtDNA sequence is identical (fig. 2*a*) over comparable sequenced regions, to that inferred from the previously published partial data for different strains of this species (Akhmanova et al. 1998; de Graaf et al. 2011), although the levels of per gene sequence identity are relatively low (24.6–93.5%, mean = 52.2%, at the amino acid level). The absence of *rps13* and a second copy of *rps4* from the previously published partial *N. ovalis* data (de Graaf et al. 2011) may be due to the incomplete nature of that data set, as the two genes are next to each other in the new *N. ovalis* sequence (fig. 2*b*). Interestingly, a tandem 34-bp 12-repeat sequence reported to be present in the middle of the previously published partial (41,666 bp) *N. ovalis* mtDNA sequence (NCBI accession: GU057832.1) (de Graaf et al. 2011) was not identified in our new mtDNA sequence. However, the published gene sequences on either side of the repeat are syntenous with the *N. ovalis* mtDNA sequence from the present study. Although the repeat section in the previously published *N. ovalis* mtDNA sequence (de Graaf et al. 2011) lacks any significant similarity to the putative telomeric repeats in the new *N. ovalis* mtDNA sequence from the present study, it is similar in length. At present it is not clear if the different locations of the repeat regions in the two sequences are real differences in genome organization or assembly artifacts.

We detected transcripts in the *C. porcatum* RNAseq data for four mitochondrial protein-coding genes and two mitochondrial ribosomal RNAs (fig. 2). The open reading frames (ORFs) of the four transcripts could be translated in full using the genetic code for ciliate mtDNA (NCBI genetic code 4), whereas translation using the predicted nuclear genetic code for this organism (NCBI genetic code 6) introduced premature stop codons. Phylogenetic analysis placed the putative *C. porcatum* mitochondrial ribosomal RNA genes in the expected part of the ciliate tree (fig. 1*b*) for this species (Gao et al. 2010, 2012). The difficulties we experienced in obtaining mtDNA from *C. porcatum* may reflect the low yield of starting material from these relatively small (∼30 μm in length) cells, each of which contains ∼15 hydrogenosomes (Esteban et al. 1993). For comparison, each larger cell (∼110 μm in length) of *M. contortus* has been estimated to contain thousands of hydrogenosomes (Finlay and Fenchel 1989).

### Gene Retention and Loss in Hydrogenosome Genomes

The coding capacity of mtDNA in aerobic mitochondria is focused upon proteins needed for the ETC complexes (complexes I–IV and F_0_F_1_ ATP-synthase), as well as some of the components needed for their translation by mitochondrial ribosomes, including ribosomal proteins, tRNAs, and the large and small ribosomal RNAs (Gray 2012). The longest and potentially most complete mtDNA sequences from the present study, for *M. contortus*, *M. es*, and *N. ovalis* (de Graaf et al. 2011), contain genes encoding subunits of complex I, mitochondrial ribosomal proteins, rRNAs, and tRNAs (fig. 2), suggesting that the main role of the mtDNA of these species is to encode proteins required to make complex I. All three species appear to have lost genes that are typically encoded by aerobic ciliate mtDNA for complexes III–V (fig. 2), which are responsible for the final stages of aerobic respiration including ATP production. The loss of these complexes appears to be a common feature (Stairs et al. 2015) of the reductive evolution of mitochondrial function in microbial eukaryotes adapting to life under low oxygen conditions (hypoxia).

Comparison of the mtDNA from *M. contortus*, *M. es*, and *N. ovalis*, which are all members of the class Armophorea (Lynn 2008), reveals relatively little synteny of gene order (fig. 2*b*). This contrasts with the mtDNA of closely related aerobic ciliates, including the oligohymenophoreans *Tetrahymena thermophila* and *Paramecium aurelia* (Burger et al. 2000) and the spirotrichs *Sterkiella histriomuscorum* and *Euplotes minuta* (de Graaf et al. 2009; Swart et al. 2012), which have conserved large regions of colinear gene order. It seems possible that the mtDNA rearrangements we observe among armorphorids are associated with reductive gene loss during adaptation to life under hypoxic conditions.

A total of 12,396 bp of mtDNA sequence was recovered from *M. striatus*, in several short contigs and transcripts. These partial data include genes for complex I and ribosomal components (fig. 2*a*) and includes a gene for Rps13, a ribosomal protein commonly found in the mtDNA of aerobes but not detected for the other Armophorea (fig. 2*a*). The differences in gene content for individual Armophorea suggest that their last common anaerobic ancestor had a more complete mitochondrial genome than the contemporary species we sampled.

The transcriptomics data for mtDNA genes from *C. porcatum* include genes for two ribosomal proteins, two complex I proteins and, uniquely among the species we investigated, a gene for a putative F_0_F_1_ ATP-synthase protein, Ymf66, which is also found in some aerobic ciliates (supplementary fig. 1, Supplementary Material online). Proteomic data for the F_0_F_1_ ATP-synthase complex from *Te. thermophila* (Nina et al. 2010) suggest that Ymf66 is a divergent homolog of the F_0_-subcomplex subunit *a* (also known as Atp6). In particular, it shares a conserved arginine residue, embedded in a predicted transmembrane helix, which is thought to be essential for the function of F_0_-subcomplex subunit *a* (Nina et al. 2010). Ymf66 appears to be well conserved in the Oligohymenophorea, the ciliate class that includes *Cyclidium* (Gao et al. 2010, 2012), and divergent copies of the gene for this protein are present in the mtDNA of the aerobic spirotrichs *Sterkiella histriomuscorum* (Swart et al. 2012) (NCBI accession: AEV66695) and *Euplotes crassus* (de Graaf et al. 2009) (NCBI accession: ACX30986).

### Ciliate Hydrogenosomes Show Different Degrees of Reductive Evolution

Most mitochondrial proteins in aerobic ciliates are encoded by the macronuclear genome (the somatic, polyploid genome of ciliates that is transcribed to produce functional proteins) and are synthesized by cytosolic ribosomes before being targeted to mitochondria (Smith et al. 2007). To identify nuclear-encoded mitochondrial genes to complement the new organelle genome data, we analyzed the single-cell transcriptome data sets (supplementary table 1, Supplementary Material online) generated for *C. porcatum*, *M. contortus*, and *P. frontata* in detail. Proteins were predicted as functioning in hydrogenosomes either based on their inferred homology with mitochondrial proteins from related organisms, including the ciliate with the best-studied mitochondria, *Te. thermophila* (Smith et al. 2007), or on the presence of mitochondrial-targeting signals (MTS) (predicted as described in Materials and Methods). The combined data, with the caveat that they are still likely to provide incomplete coverage of individual proteomes, provide insights into the similarities and differences between hydrogenosomes from three phylogenetically distinct anaerobic ciliates. The smaller single-cell transcriptome data sets (supplementary table 1, Supplementary Material online) generated for *N. ovalis*, *M. es*, *M. striatus*, and *T. finlayi* were used to identify putative hydrogenosome proteins including FeFe-hydrogenase, pyruvate:ferredoxin oxidoreductase (PFO), pyruvate:NADP^+^ oxidoreductase (PNO) and the 24- and 51-kDa subunits of complex I. These protein sequences were included in phylogenetic analyses (fig. 4).

### Hypoxia-Driven Reductive Evolution of the Mitochondrial ETC

**Fig. 3.**
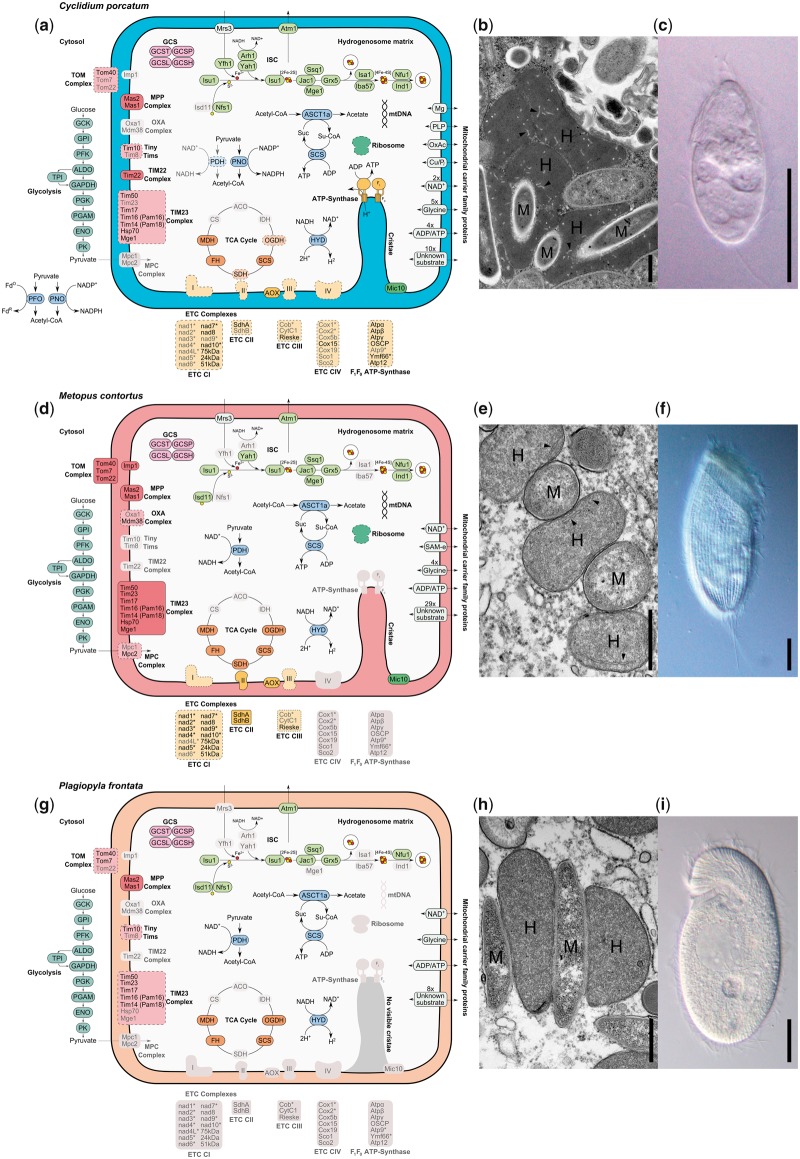
(*a*, *d*, and *g*) Metabolic maps of the hydrogenosomes from *Cyclidium porcatum*, *Metopus contortus*, and *Plagiopyla frontata*, reconstructed based on molecular data sets. The mitochondrial proteins that are shown either were detected from these three species or are present in the ciliate with the best-characterized mitochondria, *Tetrahymena thermophila* (Smith et al. 2007). Complexes for which all subunits were identified are outlined by a solid line, complexes for which some of the total subunits were identified are outlined by a dashed line, and complexes for which no subunits were identified have no outline and are colored gray. Proteins that are depicted within the hydrogenosome matrix in the metabolic maps were determined as functioning inside the hydrogenosomes, either on the basis of them having predicted N-terminal MTS or because homologs of these proteins are only found inside mitochondria in other organisms. Proteins typically encoded by mtDNA in ciliates are labeled (*). For abbreviations, see supplementary table 2, Supplementary Material online. (*b*, *e*, and *h*) TEM images for *C. porcatum* (*b*), *M. contortus* (*e*), and *P. frontata* (*h*), showing hydrogenosomes (H) and methanogenic endosymbionts (M). Scale bars represent 0.5 μm. Visible cristae within the hydrogenosomes of *C. porcatum* (*b*) and *M. contortus* (*e*) are indicated by black arrowheads. (*c*, *f*, and *i*) DIC images of living unfixed cells for *C. porcatum* (*c*), *M. contortus* (*f*), and *P. frontata* (*i*). Scale bars represent 20 μm.

In aerobic mitochondria, complexes I and II of the ETC reduce ubiquinone generating ubiquinol, which is reoxidized by complex III and the electrons transferred to O_2_ via complex IV. This regeneration of ubiquinone is important for maintaining the activity of complex I. Complexes I, III, and IV also pump protons across the inner mitochondrial membrane, generating a proton gradient that can be used by the F_0_F_1_ ATP-synthase of complex V to make ATP, as well as supporting protein import into the organelle. Our data suggest that the ETC has been reduced to different degrees in *C. porcatum* and *M. contortus* and completely lost, along with the mitochondrial genome, in *P. frontata* (fig. 3).

The subunits of complex I can be divided into three functionally and structurally distinct subcomplexes or modules (Hunte et al. 2010). They comprise the membrane-embedded, proton-pumping P-module (Nad1–Nad6 subunits), the ubiquinone-reducing Q-module (Nad7–Nad10 subunits), and the peripheral, hydrophilic, NADH-dehydrogenase N-module (73-, 24-, and 51-kDa subunits) (Hunte et al. 2010). *Metopus contortus* appears to have an almost complete complex I with only the Nad4L and Nad6 subunits not detected. These two subunits are typically encoded by mtDNA, and while they form part of the P-module, they are distinct from the antiporter-like subunits, Nad2, Nad4, and Nad5 (fig. 2*a*), which directly pump protons (Hunte et al. 2010). A similarly complete complex I was previously inferred for *N. ovalis*, and inhibitor studies for this species have shown that this is responsible for generating the hydrogenosome membrane potential (Boxma et al. 2005; de Graaf et al. 2011). We identified all three nuclear-encoded subunits of the N-module for *C. porcatum*, and three subunits of the Q-module. Given that most of the missing complex I subunits are typically encoded by mtDNA, for which we have little *C. porcatum* data, we speculate that this species has also retained a functional complex I. Although *P. frontata* appears to have lost all of the proton-pumping ETC complexes including complex I, a membrane potential needed to support protein import might be generated (Klingenberg and Rottenberg 1977) by the electrogenic exchange of ADP for ATP across the inner hydrogenosome membrane. We identified members of the mitochondrial carrier family (MCF) of inner membrane transport proteins, which could potentially mediate exchange of ADP for ATP, for all three species including *P. frontata*.

Under aerobic conditions, mitochondrial complex II oxidizes succinate to fumarate, transferring electrons to flavin adenine dinucleotide (FAD) that can be used to reduce ubiquinone to ubiquinol. We detected the catalytic subunit of complex II (SdhA) for *C. porcatum*, and both SdhA and SdhB were detected for *M. contortus*. Nuclear genes for these two proteins were also previously identified for *N. ovalis* (Boxma et al. 2005; de Graaf et al. 2011). In the absence of complexes III and IV, ubiquinone can be regenerated from ubiquinol by complex II acting in reverse as a fumarate reductase, using electrons from ubiquinol to convert fumarate into succinate (Tielens et al. 2002). The tricarboxylic acid (TCA) cycle enzymes citrate synthase (CS), aconitase (ACO), and isocitrate dehydrogenase (IDH) are not needed for ubiquinone regeneration by this route and appear to have been lost by all three species. The products of previous metabolic labeling experiments for *N. ovalis* (Boxma et al. 2005) are consistent with succinate production by fumarate reduction and also suggest that the TCA cycle is incomplete for this species.

It has previously been suggested that the fumarate reductase activity of *N. ovalis* complex II is used to regenerate rhodoquinone rather than ubiquinone (Boxma et al. 2005; Hackstein et al. 2008). Rhodoquinone has a lower redox potential than ubiquinone and hence may be more suited for transferring electrons to fumarate (Van Hellemond et al. 1995; Tielens et al. 2002). The methyltransferase protein RquA is used to convert ubiquinone into rhodoquinone during biosynthesis (Stairs et al. 2018) and was previously detected in genomic and transcriptomic data generated from five aerobic heterotrich ciliates (Stairs et al. 2018). This suggests that aerobic ciliates may also be able to use rhodoquinone under some conditions. However, RquA was not detected in the data for *C. porcatum* or *M. contortus*, nor was it previously reported for *N. ovalis*, despite rhodoquinone being detected in this species (de Graaf et al. 2011; Stairs et al. 2018).

We detected genes for alternative oxidase (AOX) in *C. porcatum* and *M. contortus*, which could potentially be used to regenerate ubiquinone or rhodoquinone (Tielens et al. 2002), using the small amounts of O_2_ found in hypoxic habitats as an electron acceptor. Consistent with this possibility, it has previously been shown that the microaerophilic scuticociliate *Philasterides dicentrarchi* expresses AOX under hypoxic conditions (Mallo et al. 2013). AOX has also been detected in some aerobic ciliates including *Tetrahymena* (Young 1983). In this case, it is thought that AOX facilitates the continued activity of complex I by providing an overflow for electrons when the complex III-cytochrome-complex IV section of the ETC is saturated, or when cellular requirements for ATP are low (Young 1983). The anaerobic human gut parasite *Blastocystis* also has an AOX and a partial ETC consisting of complexes I and II (Tsaousis et al. 2018). In *Blastocystis*, it is suggested that AOX provides a mechanism to cope with fluctuations in environmental O_2_ concentration (Tsaousis et al. 2018). Exposure of anaerobes to O_2_ is thought to cause an increased production of toxic reactive oxygen species (Fenchel and Finlay 2008), so it is possible that AOX in *C. porcatum* and *M. contortus* might also help to mitigate these effects (Maxwell et al. 1999).

The Rieske protein was the only subunit of complex III detected for *C. porcatum* and *M. contortus* (fig. 3). Rieske protein normally catalyzes the oxidation of ubiquinol, with the electrons transferred to cytochrome c via the catalytic subunits CytC1 and Cob (Iwata et al. 1996). Rieske proteins contain a [2Fe-2S] cluster binding domain, which is present in the homologs detected for *C. porcatum* and *M. contortus*. This suggests that the Rieske proteins of these species are under selection to maintain key functional residues and hence may have retained a role in electron transfer.

We detected several components of the F_1_F_0_ ATP-synthase (complex V) for *C. porcatum* that gave best blast hits to homologs from other Oligohymenophorea. These include the core catalytic subunits Atpα and Atpβ and the central stalk subunit Atpγ, all of which are part of the F_1_ subcomplex (Davies et al. 2012; Vinothkumar et al. 2016). We also detected the peripheral stalk oligomycin sensitivity conferring protein subunit (Giorgio et al. 2018) and an assembly factor Atp12 (Pícková et al. 2005). Many of the protein subunits of the F_0_-subcomplex in model eukaryotes have not been identified in ciliates (Smith et al. 2007; Nina et al. 2010). Exceptions include the putative F_0_-subcomplex protein, Ymf66 (discussed above) and the Atp9 subunit (also known as subunit *c*), both of which are encoded by mtDNA (Swart et al. 2012). The Atp9 subunit forms the membrane-embedded pore of the complex, and while it was not identified in the limited data for *C. porcatum*, the detection of nuclear-encoded subunits of F_1_F_0_ ATP-synthase common to other ciliates (Smith et al. 2007), suggests that *C. porcatum* may also possess Atp9. Based on these data, it appears possible that *C. porcatum*, uniquely among the anaerobic hydrogenosome-containing ciliates investigated, has a functional F_1_F_0_ ATP-synthase that can make ATP using the proton gradient generated by complex I.

### The Absence of Cristae Correlates with Loss of the ETC

Transmission electron microscopy (TEM) images (fig. 3) of hydrogenosomes from *C. porcatum* and *M. contortus* confirm earlier reports for the presence of cristae in these species (Finlay and Fenchel 1989; Esteban et al. 1993, 1995) and the absence of cristae in the hydrogenosomes of *P. frontata* (Embley and Finlay 1994). The mitochondrial contact site and cristae organising system (MICOS) complex is involved in the formation of cristae junctions and although two subunits of this complex, Mic10 and Mic60, are generally well conserved among eukaryotes (Muñoz-Gómez et al. 2015), only Mic10 was previously detected in ciliates (Muñoz-Gómez et al. 2015; Huynen et al. 2016). Consistent with its functional role in cristae formation, we also detected Mic10 in the data for *C. porcatum* and *M. contortus* but not in the data for *P. frontata*.

### Fe/S Cluster Biogenesis in Ciliate Hydrogenosomes

A role in Fe/S cluster biosynthesis is currently thought to be the most conserved biosynthetic function of mitochondrial homologs, and it is the sole biosynthetic function of the highly reduced genome-lacking mitochondrion (mitosome) of Microsporidia (Goldberg et al. 2008; Freibert et al. 2017). The iron sulfur cluster (ISC) pathway is used to make the [2Fe-2S] and [4Fe-4S] clusters required for maturation of mitochondrial Fe/S apoproteins (Lill 2009; Freibert et al. 2017). Previous work on *N. ovalis* detected mitochondrial ferredoxin but no other ISC pathway protein in the limited data available for this species (de Graaf et al. 2011). By contrast, we detected almost complete ISC pathways for *C. porcatum*, *M. contortus*, and *P. frontata* (fig. 3), consistent with the detection of mitochondrial Fe/S proteins including ferredoxin, SdhB, and several subunits of complex I. The FeFe-hydrogenase used to make H_2_ is also an Fe/S cluster-containing protein (Akhmanova et al. 1998) that, like known nuclear-encoded mitochondrial Fe/S proteins (Lill 2009), is probably imported into the hydrogenosome as an unfolded apoprotein lacking Fe/S clusters. The ciliate enzymes contain predicted MTS, and the close juxtaposition we observe (fig. 3*b*, *e*, and *h*) between endosymbiotic hydrogen-utilizing methanogens (supplementary fig. 5, Supplementary Material online) and the hydrogenosomes of each species (Bruggen et al. 1984; Embley and Finlay 1994; Lind et al. 2018) further support an intraorganelle location for the ciliate FeFe-hydrogenases. Some eukaryotes, including *Chlamydomonas* and *Trichomonas*, are thought to use a distinct set of enzymes (HydE, HydF, or HydG) for the maturation of FeFe-hydrogenase (Meyer 2007; Hug et al. 2010). However, since none of these proteins were detected in our data, it appears possible that Fe/S clusters are added to the apo-hydrogenase after protein import, by the existing mitochondrial ISC machinery.

In yeast and other eukaryotes, the mitochondrial ISC pathway provides a critical substrate for the cytosolic biosynthesis of essential cytosolic and nuclear Fe/S proteins including DNA polymerase (Paul and Lill 2015; Freibert et al. 2017). The export of this substrate is mediated in yeast by the mitochondrial ABC transporter Atm1 (Paul and Lill 2015). We detected homologs of Atm1 in all three species (fig. 3), suggesting that ciliate hydrogenosomes have retained this essential role in cellular Fe/S protein biosynthesis.

### Ciliate Hydrogenosomes Contain Multiple Members of the MCF of Transport Proteins

The metabolism of aerobic mitochondria is sustained by the transfer of substrates and metabolites across the inner mitochondrial membrane by dedicated members of the MCF of transport proteins (Kunji 2004). Eukaryotes with canonical mitochondria typically contain between 35 and 55 MCF transporters (Kunji 2004), with 53 MCF detected for the aerobic ciliate *Tetrahymena* (Smith et al. 2007). By contrast, the genome of *Trichomonas vaginalis* (Carlton et al. 2007), which has a genome-lacking hydrogenosome, has only five genes annotated as MCF proteins, and Microsporidia have lost all MCF from their minimal mitochondria (mitosomes) (Goldberg et al. 2008; Tsaousis et al. 2008; Hjort et al. 2010; Freibert et al. 2017). We detected 26 MCF for *C. porcatum*, 37 for *M. contortus*, and 11 for *P. frontata* (fig. 3 and supplementary table 3 and fig. 2, Supplementary Material online), consistent with the retention of diverse mitochondrial functions by the hydrogenosomes of these ciliates. Putative substrates for the ciliate MCF were inferred from phylogenetic analyses when they clustered with characterized MCF from *Saccharomyces cerevisiae* with bootstrap support values of 80% or over (supplementary table 3 and fig. 2, Supplementary Material online).

We detected putative ADP/ATP, Glycine, and NAD^+^ transporters for all three ciliates. The ADP/ATP transporters are related to yeast homologs that can import and export ATP and thus could potentially support ATP-requiring reactions inside the hydrogenosomes (fig. 3). All three ciliates also have complete glycolytic pathways that could provide the cytosolic ATP used for import. ADP/ATP transporters were also reported previously for *N. ovalis* (Voncken et al. 2002; Hackstein et al. 2008). The detection of putative glycine transporters is consistent with detection of the glycine cleavage pathway, which plays a role in mitochondrial amino acid metabolism and nucleotide biosynthesis. The P-protein of the glycine cleavage pathway is dependent on the coenzyme pyridoxal 5′-phosphate, and we detected a putative pyridoxal 5′-phosphate transporter in *C. porcatum*. Components of the glycine cleavage pathway were previously detected for *N. ovalis* (de Graaf et al. 2011). The putative NAD^+^ transporters detected could potentially provide NAD^+^ used in pyruvate decarboxylation (fig. 3). Homologs of the yeast iron transporter Mrs3 were detected for *C. porcatum* and *M. contortus* (fig. 3) and putative oxaloacetate, copper/phosphate, and Mg^2+^ carriers were detected for *C. porcatum.* The additional ciliate MCF proteins detected were not assigned a putative substrate because they did not cluster strongly with characterized yeast transporters. However, since most of them cluster strongly with orthologs from *Tetrahymena*, it seems likely that they sustain functions that are conserved between hydrogenosomes and the mitochondria of this aerobic ciliate.

### Hydrogenosome Pyruvate Metabolism and ATP Production by Substrate Level Phosphorylation

We detected putative hydrogenosomal enzymes for pyruvate decarboxylation for all three ciliates. In yeast, pyruvate is translocated into mitochondria by the mitochondrial pyruvate carrier complex (Bricker et al. 2012; Herzig et al. 2012), which consists of two subunits, Mpc1 and Mpc2. Homologs of both subunits are present in the *Te. thermophila* genome and we detected a homolog of Mpc2 in the data for *M. contortus*, but not for *C. porcatum* or *P. frontata*. In *Trichomonas*, malic enzyme is used to convert hydrogenosomal malate into pyruvate (Mertens 1993). The malate is imported into hydrogenosomes using the malate/aspartate shuttle, which includes malate dehydrogenase. We identified malic enzyme and malate dehydrogenase from *P. frontata*, suggesting that it could potentially supply pyruvate using this pathway. We did not detect either enzyme in the data for *C. porcatum* and *M. contortus.*

The mitochondrial pyruvate dehydrogenase complex (PDH) typically catalyzes the oxidative decarboxylation of pyruvate, yielding acetyl-CoA, NADH, and CO_2_. We detected all four subunits (E1α, E1β, E2, and E3) of the PDH complex (PDH) for *M. contortus* and *P. frontata*, suggesting that these species, like *N. ovalis* (de Graaf et al. 2011), have retained a functional PDH. We also detected the dihydrolipoyl dehydrogenase E3 subunit of PDH for *C. porcatum*, but since this also functions as part of the oxoglutarate dehydrogenase (OGDH) complex (Massey et al. 1960) and the glycine cleavage system (Klein and Sagers 1967) (fig. 3), it may not indicate the presence of a complete PDH complex. Instead, our data suggest that *C. porcatum* uses a different enzyme to decarboxylate pyruvate and make acetyl-CoA and NADH, because we detected four homologs of the O_2_-sensitive enzyme PFO. One of these is a classical PFO that is predicted to use ferredoxin as an electron acceptor (Gorrell et al. 1984). The other three genes code for a PFO-fusion protein called PNO (Nakazawa et al. 2000; Rotte et al. 2001) that contains a NADPH-cytochrome P450 reductase domain potentially capable of reducing NADP^+^ (Inui et al. 1984). Two of the *C. porcatum* PNOs contain putative MTS (supplementary data 1, Supplementary Material online), suggesting that they decarboxylate pyruvate using NADP^+^ inside *C. porcatum* hydrogenosomes. The lack of MTS for the PFO and remaining copy of PNO suggests that they function in the ciliate cytosol (fig. 3).

The hydrogenosomes of *Trichomonas vaginalis* (Hrdý et al. 2007) produce ATP from acetyl-CoA by substrate-level phosphorylation using acetate:succinate CoA transferase (ASCT) (van Grinsven et al. 2008) and succinyl CoA synthetase ((Jenkins et al. 1991). Homologs of ASCT and succinyl CoA synthetase were identified for all three ciliates and previously for *N. ovalis* (de Graaf et al. 2011), suggesting that ciliate hydrogenosomes may also make ATP by substrate-level phosphorylation (fig. 3).

### A Mitochondrial Protein Import System in Ciliate Hydrogenosomes

Nuclear-encoded mitochondrial proteins are imported into mitochondria using a multicomponent system that has evolved to deliver proteins to different mitochondrial compartments and membranes (Dudek et al. 2013). We detected components from the main mitochondrial translocase complexes (TOM40, TIM22, and TIM23) for all three ciliates (fig. 3).

We identified the Tom40 subunit of the TOM40 outer membrane translocase in data for *C. porcatum*, *M. contortus*, and *P. frontata*, the Tom7 subunit for *M. contortus* and *P. frontata*, and the Tom22 subunit for *M. contortus*. We also detected a homolog of Imp1, the inner membrane peptidase used to process N-terminal MTS, for *M. contortus*. Once through the outer membrane, proteins destined for the mitochondrial matrix or inner membrane are processed separately by either the TIM23 or TIM22 translocase complexes, respectively. Subunits of the TIM23 complex were detected for all three species (fig. 3), consistent with the detection of proteins predicted to have MTS and to function in the matrix of hydrogenosomes (supplementary data 1, Supplementary Material online). We also detected both subunits (Mas1 and Mas2) of the MPP complex that is used to cleave mitochondrial MTS as they enter the mitochondrial matrix via TIM23 (Jensen and Dunn 2002), for all three ciliates. Tim22 is the only subunit of the TIM22 complex that has so far been detected in *Te. thermophila* (Smith et al. 2007) and we detected a Tim22 homolog for *C. porcatum*, but not for *M. contortus* or *P. frontata*. Hydrophobic proteins are typically guided to the TIM22 complex by the Tiny Tim chaperones (Dolezal et al. 2006) and we detected Tim10 for *C. porcatum* and *P. frontata*.

### Origin of the Ciliate Multidomain FeFe-Hydrogenase

**Fig. 4.**
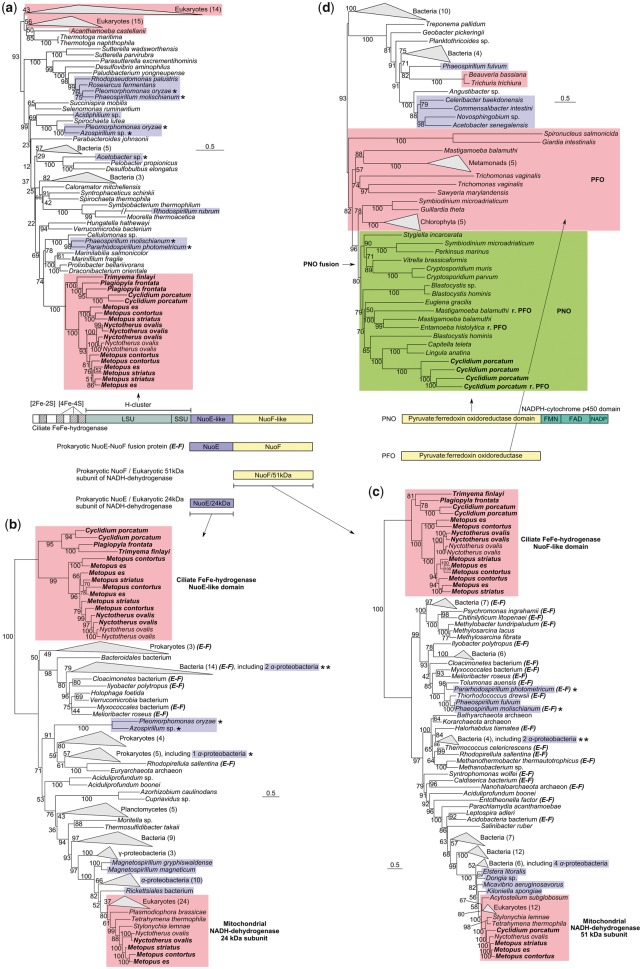
Protein domain structures of key anaerobic metabolism enzymes and corresponding phylogenies, inferred by IQ-TREE using the LG + C60 models: (*a*) the H-cluster, consisting of large (LSU) and small (SSU) subunit domains, of FeFe-hydrogenase; (*b*) NuoE-like domain of FeFe-hydrogenase from ciliates, bacterial NuoE, and eukaryotic 24-kDa subunits of NADH-dehydrogenase; (*c*) NuoF-like domain of FeFe-hydrogenase from ciliates, bacterial NuoF, and eukaryote 51-kDa subunits of NADH-dehydrogenase; eukaryotes are highlighted in red. (*d*) PFO (eukaryotes highlighted in red) and PFO-like domains of eukaryotic PNO (highlighted in green), the branch where the PNO fusion is likely to have occurred is shown. In all phylogenies, α-proteobacteria sequences are highlighted in blue and sequences obtained in the present study are shown in bold. Prokaryotic sequences that are NuoE (*b*) or NuoF (*c*) domains of NuoE-NuoF fusion proteins are indicated ***(E-F)***. Sequences that were also investigated by Esposti et al. 2016 are labelled (*). Support values, displayed as percentages, were generated from 1,000 ultrafast bootstrap replicates for each tree. Scale bars represent the number of substitutions per site. A small number of the FeFe-hydrogenase sequences from ciliates in the present study were truncated and lacked NuoE or NuoF domains, as they were encoded by incompletely sequenced transcripts. In such cases, only the H-cluster domain of these proteins could be analyzed (*a*), hence why they are not present in (*b*) and (*c*).

Previous phylogenetic analyses of eukaryotic FeFe-hydrogenases (Horner et al. 2000; Davidson et al. 2002; Embley et al. 2003; Meyer 2007; Hug et al. 2010; Greening et al. 2016) have recovered most enzymes in a large cluster (called clade A in Hug et al. [2010]) that also includes sequences from diverse bacteria. Some bacterial FeFe-hydrogenases are heteromeric complexes formed by two separately encoded proteins, referred to as the large and small FeFe-hydrogenase subunits (Nicolet et al. 1999). By contrast, all of the FeFe-hydrogenases in clade A have a different structure whereby the large and small subunits are encoded together as two subdomains (together forming the H-cluster active site) of the same protein. In the present study, we included representative sequences from clade A and based our analysis (fig. 4*a* and supplementary fig. 3*a*, Supplementary Material online) upon the conserved H-cluster of FeFe-hydrogenase sequences.

The FeFe-hydrogenases of anaerobic ciliates formed a single strongly supported cluster separate from the other eukaryotes (fig. 4*a*). With the exception of *T. finlayi*, we identified multiple FeFe-hydrogenase paralogs for each ciliate, demonstrating that gene duplication is a feature of ciliate FeFe-hydrogenase evolution. Relationships between ciliate FeFe-hydrogenases are consistent with published ciliate relationships (Gao et al. 2016) (fig. 1), in that the basal split is between *Metopus*/*Nyctotherus* on one side and *Plagiopyla*/*Trimyema/Cyclidium* on the other. This topology suggests that the sampled anaerobic ciliates, which are not monophyletic to the exclusion of ciliates with mitochondria (Embley et al. 1995; Gao et al. 2016) (fig. 1*a*), inherited genes for FeFe-hydrogenase from a common ancestor shared with aerobic ciliates.

Although some individual groups in the FeFe-hydrogenase tree (fig. 4*a*) are strongly supported, the backbone of the tree and hence the relationships between groups are only weakly supported by bootstrapping. The low bootstrap support values in FeFe-hydrogenase trees have been noted before (Embley et al. 2003; Hug et al. 2010), with sequence saturation thought to be a contributing factor to the lack of resolution (Horner et al. 2000). To investigate further the strength of support for an independent origin of ciliate sequences, we evaluated whether alternative topological rearrangements under the best-fitting LG + C60 (Le and Gascuel 2008) model could be rejected (*P* < 0.05) using the approximately unbiased (AU) likelihood-based test (Shimodaira 2002). The following constraints were evaluated: 1) all eukaryotic FeFe-hydrogenases (plus the bacterium *Thermotoga*) constrained as a single group (*P* value = 0.424), 2) constraining the ciliate and *Vitrella brassicaformis* (an alveolate, like ciliates) FeFe-hydrogenases together (*P* value = 0.466), and 3) constraining the ciliate and *V. brassicaformis* FeFe-hydrogenases within the main group of eukaryotic sequences (plus the bacterium *Thermotoga*) (*P* value = 0.246). The results of these analyses reveal that, although a single separate origin of ciliate FeFe-hydrogenases is favored by the maximum likelihood tree, none of the alternative topologies we tested were significantly rejected using the AU test at *P* < 0.05.

A separate single origin for the ciliate FeFe-hydrogenase is also supported by a common unique multidomain structure. The ciliate FeFe-hydrogenases possess two C-terminal domains with similarity to the NuoE/HoxE and NuoF/HoxF subunits of bacterial NADH-dehydrogenases/NADH-dependent NiFe-hydrogenases (Horner et al. 2000; Boxma et al. 2007). The addition of the NuoE-like and NuoF-like domains to an FeFe-hydrogenase appears to be ciliate specific and it is not a feature of the FeFe-hydrogenase of *V. brassicaformis*. The NuoE-like and NuoF-like domains would potentially allow the ciliate FeFe-hydrogenases to couple the oxidation of NADH to H_2_ production (Akhmanova et al. 1998; Horner et al. 2000). Separate phylogenetic analyses of the NuoE-like and NuoF-like domains recovered both sets of ciliate sequences as distinct clusters (fig. 4*b* and *c*) in trees dominated by bacterial sequences. The observed topological congruence for individual components of the ciliate FeFe-hydrogenase, suggests that they were already together as a functional unit in the ancestral enzyme. Fused NuoE and NuoF subunits encoded by a single gene are also a feature of some bacterial NADH-dehydrogenases (NuoE and NuoF sequences labeled ***(E-F)*** in fig. 4*b* and *c*).

The NuoE-like and NuoF-like domains of the ciliate FeFe-hydrogenase are distinct from the homologous 24- and 51-kDa subunits of mitochondrial NADH-dehydrogenase from the same ciliates. The latter cluster with mitochondrial proteins from aerobic eukaryotes and orthologs from alphaproteobacteria, consistent with their origin from the mitochondrial endosymbiont. In agreement with some analyses (Horner et al. 2000; Boxma et al. 2007) but not others (Esposti et al. 2016), we recovered no topological support for a specific alphaproteobacterial origin for the ciliate NuoE-like and NuoF-like domains (fig. 4*b* and *c* and supplementary fig. 3*b* and *c*, Supplementary Material online). Additional analyses of a broader sample of prokaryotic NuoE-NuoF fusion proteins (supplementary fig. 4, Supplementary Material online) also failed to provide any support for an alphaproteobacterial ancestry of the ciliate NuoE-like and NuoF-like domains.

Our analyses suggest that the unique multidomain FeFe-hydrogenase used to make H_2_ in the hydrogenosomes of all three ciliates was inherited from the last common ancestor of the species sampled. Based on our data and the ciliate tree topology, it seems likely that the last common ancestor possessed a mitochondrion that was capable of oxidative phosphorylation, posing the question of how FeFe-hydrogenase, a notoriously oxygen-sensitive enzyme (Zimorski et al. 2019) was retained by, and inherited from, that ancestor. The answer may lie with ciliate ecology and the apparent ease by which diverse ciliates can tolerate and adapt to low oxygen conditions (Finlay 1981; Bernard and Fenchel 1996). Ciliates are often very abundant at the oxic/anoxic boundary where they thrive as the main particulate feeders on the rich microbial populations such habitats support (Finlay 1981; Bernard and Fenchel 1996). Under low oxygen conditions the NuoE-like and NuoF-like domains of ciliate FeFe-hydrogenase would help to maintain cellular redox balance by oxidizing NADH and regenerating NAD^+^ for glycolysis. This metabolic flexibility could provide a selective advantage for the retention of the FeFe-hydrogenase. It also generates the testable prediction that other anaerobic ciliates that contain hydrogenosomes (Fenchel and Finlay 1995) will be found to use the same type of FeFe-hydrogenase. An early acquisition and retention of FeFe-hydrogenase among ciliates would have also made them a commonly encountered endosymbiotic niche for anaerobic methanogens living in the same habitats (Fenchel and Finlay 1995). The acquisition of endosymbiotic methanogens consuming H_2_ would in turn provide ciliates with an additional means of maintaining redox balance in O_2_-depeleted environments (Fenchel and Finlay 1992), enhancing host fitness (Fenchel and Finlay 1991) and facilitating the loss of genes for the later O_2_-dependent stages of the ETC that we observed in our data.

The source(s) of the FeFe-hydrogenases used to make H_2_ in eukaryotic hydrogenosomes more generally have been much debated (Embley et al. 1997; Martin and Müller 1998; Muller et al. 2012; Stairs et al. 2015). The main ideas discussed are a single common origin from the alphaproteobacterial mitochondrial endosymbiont (Martin and Müller 1998; Martin et al. 2015; Esposti et al. 2016), or multiple independent origins in different anaerobic eukaryotes thorough lateral gene transfer (LGT) from bacteria occupying the same anaerobic habitats (Stairs et al. 2015). Although our trees cannot exclude the possibility of a common origin for the eukaryotic FeFe-hydrogenases according to the results of AU tests, they provide no topological support for an alphaproteobacterial origin for eukaryotic sequences as a whole, or the ciliate FeFe-hydrogenases in particular. Sequences from alphaproteobacteria were dispersed throughout the tree in clusters containing a mixture of different bacteria, suggesting that LGT, gene duplication, and gene loss could have all played a role in the evolution of bacterial FeFe-hydrogenases (Horner et al. 2000; Embley et al. 2003; Esser et al. 2007; Hug et al. 2010). The significant problems for identifying or eliminating the mitochondrial endosymbiont as a source of eukaryotic genes, like FeFe-hydrogenase (or PFO see below), that such genome fluidity presents, have already been discussed in detail elsewhere (Embley and Martin 2006; Esser et al. 2007).

### PFO and PNO in *C. porcatum*

Some anaerobic eukaryotes use the oxygen-sensitive enzyme PFO for pyruvate oxidation instead of PDH, in either the cytosol (e.g., *Giardia*) or the hydrogenosome (e.g., *Trichomonas*) (Muller et al. 2012). Like FeFe-hydrogenase, the origin of eukaryotic PFO has been debated with the same possible sources including the mitochondrial endosymbiont or separate LGTs proposed (Martin and Müller 1998; Embley and Martin 2006; Martin et al. 2015; Stairs et al. 2015). Previous studies have been unable to reject monophyly of most eukaryotic PFO sequences including PFO-fusion proteins like PNO, but have nevertheless recovered relationships among eukaryotes and prokaryotes that are difficult to reconcile with simple vertical inheritance (Horner et al. 1999; Rotte et al. 2001; Embley et al. 2003; Hug et al. 2010; Nývltová et al. 2015). We obtained a similar picture from our own analyses (fig. 4*d* and supplementary fig. 3*d*, Supplementary Material online). Most eukaryotic enzymes cluster together but with no clear indication from current sampling for an origin from the alphaproteobacteria or a specific bacterial group. The eukaryotic PNO sequences were recovered as a single cluster consistent with a common origin through a fusion of PFO with a NADPH-cytochrome P450 oxidoreductase module (Nakazawa et al. 2000; Rotte et al. 2001). The four *C. porcatum* sequences form a single cluster with maximum support within this group that is strongly separated from other alveolates like *Cryptosporidium* and *Vitrella* (fig. 4*d*). The PNO cluster also contains several sequences that have secondarily lost the NADPH-cytochrome P450 oxidoreductase domain and reverted to PFO, including one of the four *C. porcatum* sequences.

## Conclusions

Anaerobic ciliates provide an opportunity to investigate a rare example of the repeated hypoxia-driven reductive evolution of mitochondria into hydrogenosomes within a single taxonomic group. Our data reveal similarities and differences in the degree of gene loss in the different lineages. We detected evidence for the retention of a reduced mitochondrial genome in *Metopus* spp. and in *C. porcatum*. These data, in combination with previous work on *Nyctotherus* (Akhmanova et al. 1998; Boxma et al. 2005; de Graaf et al. 2011), suggest that the conservation of genes needed to make a functional complex I is a major driver for mitochondrial genome retention inside ciliate hydrogenosomes. Consistent with this idea, we identified nuclear genes for complex II and other proteins including AOX, that can potentially regenerate the ubiquinone needed to sustain complex I function in the absence of a complete mitochondrial ETC. *Cyclidium porcatum* is so far unique among hydrogenosome-containing ciliates in that it has retained complex V and hence is potentially capable of generating ATP using the proton gradient generated by complex I. By contrast, the hydrogenosomes of *P. frontata* have lost the mitochondrial genome and ETC in their entirety.

We detected multiple genes for MCF proteins for each species, with differences in MCF abundance for individual species consistent with different degrees of metabolic reduction. Mitochondrial pathways retained in common include a capacity for pyruvate decarboxylation and ATP production by substrate-level phosphorylation, retention of the glycine cleavage pathway, and a biosynthetic role in the maturation of cellular Fe/S proteins that are essential for cell survival. The latter appears to be the most conserved biosynthetic function for mitochondrial homologs across the eukaryotic tree (Freibert et al. 2017). The detection for each ciliate of multiple MCF genes for transporters of unknown function, but which are also conserved in the *Tetrahymena* genome, suggests that ciliate hydrogenosomes share a number of additional unidentified functions with the aerobic mitochondria of ciliates.

Our results also have relevance for ongoing and topical debates about mitochondrial biochemistry and evolution in early eukaryotes (Martin et al. 2015; Stairs et al. 2015; Spang et al. 2019). They provide an example of how metabolically flexible organelles capable of both aerobic and anaerobic biochemistry could have been maintained by microbial eukaryotes at the margins of the early oxic/anoxic world. They also highlight gene loss as a predominant mechanism by which hydrogenosomes have evolved from mitochondria in different lineages within a single phylogenetic group and suggest that horizontal and vertical inheritance can each play a role in the remodeling of mitochondrial function.

## Materials and Methods

### Growth, Isolation, and Imaging of Anaerobic Ciliates

Free-living anaerobic ciliates were obtained by collecting water and sediment from field sites in Dorset (UK). Freshwater species were isolated from a pond that forms part of East Stoke Fen (GPS 50.679064, -2.191587) and marine species were isolated from a saltwater lake in Poole Park (GPS 50.715541, -1.971177). These samples were used to partially fill 125-ml glass vials, which were topped up with enrichment medium, leaving a small headspace. A wheat grain and a small amount of dried cereal leaves were then added to each vial. The enrichment medium used to culture freshwater species was SES (soil extract with added salts) medium and to culture marine species, N75S (new cereal leaf 75% seawater) medium was used (both recipes available from Culture Collection of Algae and Protozoa: https://www.ccap.ac.uk/; last accessed October 28, 2019). Culture vials were sealed with rubber stoppers and crimped aluminum collars. The gaseous headspaces of the vials were continually flushed with N_2_ for 3 min to remove O_2_, via hypodermic needles piercing the stoppers (one needle to let gas in via a tube from a canister of compressed N_2_ and the other to let gas out). Cultures of single ciliate species were produced by transferring individual ciliate cells to preincubated vials of medium with a micropipette. The cultures were continually incubated at 18 °C and subcultured every 4–6 weeks by inoculating vials containing 70 ml of sterile media with 30 ml of a mature culture. Cells of *N. ovalis* were obtained directly from cockroaches of the species *Blaptica dubia*, acquired commercially from Cricket Express (cricketexpress.se). Cockroaches were reared in plastic boxes and fed dried dog food and fresh fruit. To extract *N. ovalis* cells, cockroaches were dissected and their hindguts removed. *Nyctotherus ovalis* cells were then isolated from the hindguts using electromigration (Hoek et al. 1999). Methods used for transmission electron microscopy (TEM) imaging ciliate cells have been described in detail previously (Lewis et al. 2018; Lind et al. 2018). In brief, this included concentrating ciliate cells by centrifugation, followed by fixation in 2.5% glutaraldehyde. Postfixation and embedding was performed by Benoît Zuber and Beat Haenni (Microscopy Imaging Center, Institute of Anatomy, University of Bern, Switzerland), as part of a commercially provided service. Differential interference contrast imaging of ciliate cells was performed using an Olympus BH-2 light microscope and photographed with a Micropublisher 3.3 RTV mounted camera (QImaging). Ciliate species were identified visually, based on their morphology, and where necessary by silver staining methods (Fernandez-Galiano 1994). These identifications were subsequently confirmed by comparing 18S sequences obtained from RNAseq data (methods described below) to sequences from the same species in NCBI databases.

### Enrichment of Hydrogenosomes and Preparation of mtDNA for Sequencing

For each species, 200 cells were isolated by micropipette and washed by three centrifugations at 400 × g, with the supernatant being replaced with sterile phosphate-buffered saline (PBS) after each wash, in order to deplete prokaryotic contaminants. For marine species, the salinity of the PBS wash buffer was adjusted with NaCl to approximately that of the enrichment medium, as measured using a bench-top osmometer. Cells were transferred to a microcentrifuge tube and lysed mechanically by hand using a sterile pestle. Macronuclei and larger cell debris were removed by centrifugation at 400 × g for 5 min and 800 µl of supernatant then transferred to a new tube, to which 200 µl of PBS was added. This step was repeated in total twice, with the aim of enriching the samples for hydrogenosomes, which was important due to the large amount of DNA contained in the ciliate macronuclei, which would otherwise dominate the data sets generated from these samples. The enriched samples were then filtered through a 5-µm syringe filter and pelleted by centrifugation at 12,000 × g for 15 min, after which the supernatant was removed. DNA from the enriched samples was amplified by multiple displacement amplification, using a Repli-G mini kit (Qiagen), and purified using a QIAamp DNA midi kit (Qiagen), according to the manufacturer’s standard protocol. DNA libraries were produced using a Nextera XT DNA Library Preparation Kit (Illumina), and sequenced using a MiSeq (Illumina), generating paired-end 250-bp reads.

### Detection of Mitochondrial Genome (mtDNA) Sequences and Prediction of ORFs and Encoded Proteins

All ciliate mtDNA sequences available from the NCBI nt database, and the proteins predicted from these sequences, were used as queries in searches against the data sets generated in the present study, using BLAST (Altschul et al. 1990) and HMMER (http://hmmer.janelia.org/). ORFs and proteins were predicted from the identified mtDNA contigs using TransDecoder (Haas and Papanicolaou 2017), with the protozoan mitochondrial genetic code (NCBI translation table 4). Contigs were identified as corresponding to ciliate mtDNA based on the proteins they encode being translated in entirety using the protozoan mitochondrial genetic code (NCBI translation table 4), which appears to be used by all ciliates studied so far (Swart et al. 2012). Conversely, translation using the macronuclear code for the corresponding ciliate introduced premature stop codons for these contigs. Additional support for this was provided by the proteins from the mtDNA contigs lacking detectable MTS and their being generally encoded by other ciliate mitochondrial DNAs.

### Generation of cDNA and Transcriptome Sequencing

Single ciliate cells from each species were isolated from cultures by pipetting and washed by transferring them twice in sterile water before isolating them in 0.5 μl volumes. These individual single-cell samples were then lysed and used to generate cDNA, according to the Smart-seq2 protocol (Picelli et al. 2014). The cDNA libraries were prepared for sequencing using a Nextera XT DNA Library Preparation Kit (Illumina) and sequenced using a HiSeq2500 (Illumina) with rapid run mode, generating paired-end 250-bp reads.

### Transcriptome Assembly

Raw reads were assembled using Trinity v2.4.0 (Grabherr et al. 2011). DNA library preparation-related sequences, primers, and low-quality sequencing data were removed using Trimmomatic (Bolger et al. 2014), within the Trinity program. The Trimmomatic settings added to the Trinity command were ILLUMINACLIP:2:30:10, LEADING:5, TRAILING:5 SLIDINGWINDOW:5:16, and MINLEN:80.

### Detection of Macronuclear-Encoded Hydrogenosome Proteins

Putative hydrogenosome proteins were detected from translated transcriptome data sets by BlastP searches, using proteins from well described mitochondrial proteomes as queries, such as the ciliate *Te. thermophila* (Smith et al. 2007), humans (Taylor et al. 2003), and yeast (Prokisch et al. 2004), as well as homologs from other species identified from BLAST searches against the NCBI nr database. Additional searches were conducted with HMMER, using hmm profiles built from custom protein alignments or Pfam domain alignments, which were downloaded from the Pfam database. Phylogenetic analyses including sequences from reference ciliates were used to confirm the identity of putative ciliate sequences. Additional evidence that the new sequences were not contaminants was provided by codon usage analysis (supplementary methods 1, table 4, and fig. 6, Supplementary Material online). Proteins were determined as containing MTS based on predictions from Mitoprot (Claros and Vincens 1996), MitoFates (Fukasawa et al. 2015), and TargetP (Emanuelsson et al. 2007).

### Genomic Assembly

Read quality of the generated data was assessed using FastQC (Andrews 2010). SeqPrep (https://github.com/jstjohn/SeqPrep) was then used to remove short reads, remove Illumina adapters, and merge overlapping paired-end reads. Low-quality bases were removed using Trimmomatic (Bolger et al. 2014) with the parameters TRAILING:20 and MINLEN:150. Paired-end reads were assembled into contigs using the SPAdes Genome Assembler (Bankevich et al. 2012) with the parameters –sc and –careful to reduce mismatches and indels.

### Phylogenetic Analyses

For phylogenetic trees inferred from both nucleotides and proteins, sequences were aligned using Muscle 3.8.31 (Edgar 2004). Poorly conserved sites from the ends of the alignments were removed manually, and any remaining ambiguously aligned sites were removed using trimAl v1.4 (Capella-Gutiérrez et al. 2009) with the -gappyout setting. 18S and 16S rRNA sequence phylogenies were inferred using the CAT + GTR models in Phylobayes MPI (Lartillot et al. 2013), running three independent MCMC chains until two had converged. Convergence was assessed using bpcomp and tracecomp that are part of the Phylobayes MPI package. Phylogenies used for the initial screening of data sets for protein homologs were inferred using the LG model (Le and Gascuel 2008) in FastTree 2.1.10 (Price et al. 2010). Further in-depth phylogenetic analyses of protein homologs were then performed using IQ-Tree 1.6.2 (Nguyen et al. 2015), with 1,000 ultrafast bootstrap replicates (Minh et al. 2013), utilizing the built-in model test option. Maximum likelihood tree searches and AU tests (Shimodaira 2002) were conducted in IQ-Tree 1.6.2, using 10,000 RELL bootstraps (Kishino et al. 1990).

## Supplementary Material

msz239_Supplementary_Data

## Notes

All sequencing data generated in the present study have been deposited in an NCBI BioProject (accession no. PRJNA542330).
