# Supplementary material for: Convergent Evolution of Hydrogenosomes from Mitochondria by Gene Transfer and Loss
Source: Mol Biol Evol. 2019 Oct 24;37(2):524–39. doi: 10.1093/molbev/msz239 (PMC6993867; doi:10.1093/molbev/msz239)
Supplement: msz239_Supplementary_Data [file msz239_supplementary_data.zip › msz239-suppl_data/Supplementary_information.pdf]

Supplementary information for manuscript "Gene loss and gene transfer in the evolution of ciliate hydrogenosomes" by Lewis et al.

Contents

Supplementary Methods

Supplementary Methods 1. Codon usage analysis for ciliate genes.

References for Supplementary Methods 1

Supplementary Tables

Supplementary Table 1. The amount of data generated for ciliates in the present study

Supplementary Table 2. Table of abbreviations used in Figure 3 (main manuscript)

Supplementary Table 3. Putative substrates of MCF proteins from *Cyclidium porcatum*, *Metopus contortus* and *Plagiopyla frontata*

Supplementary Table 4. CAI scores for ciliate FeFe-hydrogenase, PFO and PNO genes.

Supplementary Figures

Supplementary Figure 1. Phylogeny of Ymf66 from ciliates.

Supplementary Figure 2. Phylogeny of ciliate Mitochondrial Carrier Family proteins detected in the present study and homologues from *Tetrahymena thermophila* and *Saccharomyces cerevisiae.*

Supplementary Figure 3, a. Phylogeny of FeFe-hydrogenase.

Supplementary Figure 3, b. Phylogeny of mitochondrial 24 kDa subunit of NADH-dehydrogenase, prokaryotic NuoE and the NuoE-like domain of ciliate FeFe-hydrogenase.

Supplementary Figure 3, c. Phylogeny of mitochondrial 51 kDa subunit of NADH-dehydrogenase, prokaryotic NuoF and the NuoF-like domain of ciliate FeFe-hydrogenase.

Supplementary Figure 3, d. Phylogeny of PFO and PNO.

Supplementary Figure 4. A phylogeny inferred from an alignment of prokaryotic NuoE-NuoF fusion proteins and the NuoE-like and NuoF-like C-terminal domains of ciliate FeFe-hydrogenases.

Supplementary Figure 5. F420 autofluorescence of endosymbiotic methanogens.

Supplementary Figure 6. Codon usage CAI score distribution plots.

Supplementary Data (See attached files)

Supplementary Data 1. Genes and protein sequences used to reconstruct hydrogenosome metabolisms detected from (a.) *Cyclidium porcatum*, (b.) *Metopus contortus* and (c.) *Plagiopyla frontata*.

Supplementary Data 2. mtDNA sequences from *Nyctotherus ovalis*, *Metopus contortus*, *Metopus es*, *Metopus striatus* and *Cyclidium porcatum*.

Supplementary methods 1 Codon usage analysis for ciliate genes

As was the case for the datasets generated for *M. contortus*, *M. es*, *M. striatus*, *N. ovalis*, *C. porcatum*, *P. frontata* and *T. finlayi* in the present study, *de novo* transcriptome datasets from organisms grown in non-axenic, mixed-microbial cultures, will typically contain a proportion of sequences from organisms that are not the intended target of such studies. Although our cultures each contained just a single ciliate species, they also contained a mixed community of prokaryotes that provide a food source for the ciliates. Given that there are no complete macronuclear genome sequences available for the ciliates in the present study, we could not directly confirm that genes detected in our transcriptomes were of ciliate origin by checking whether they are found on genomic contigs with other ciliate genes. We therefore used an alternative approach to rule out the possibility that the proteins we detected were not contaminants from one of the prokaryotic species co-inhabiting our cultures, and confirm that they were encoded by genes found on the ciliate genomes. This involved an analysis of codon usage bias for each ciliate, against which we could compare the codon usage of individual genes. This was particularly important for those proteins that were inferred to have been acquired by lateral gene transfer and in some cases clustered with bacterial homologues in phylogenetic analyses (Fig. 4).

Codon usage profiles for all genes from the genomes of *M. contortus*, *M. es*, *M. striatus*, *N. ovalis*, *C. porcatum*, *P. frontata* and *T. finlayi* were inferred for each species separately, by extracting a subset of genes from the respective transcript datasets that could be confidently determined as being encoded by the ciliate macronuclear genomes. These subsets included all the genes that had best blastx hits with proteins from other ciliate species in searches performed against the NCBI non-redundant protein database. Using the program cusp(Rice et al., 2000) the codon usage for each gene in these subsets were assessed, and used to generate codon usage tables that represent an estimated profile of the codon usage for the total genome.

The codon usage bias for each of the genes in the subsets was then analysed by calculating codon adaptation index (CAI)(Sharp and Li, 1987) scores, according to the codon usage tables, using the program cai(Rice et al., 2000). These calculated CAI scores for were then plotted as frequency distributions for each ciliate species (Supp. Fig. 5). Each of the ciliate CAI score frequency distributions were unimodal, suggesting that the genes in the extracted subsets were all from the same genome.

We next determined whether the genes for the individual proteins that were used to reconstruct hydrogenosome metabolisms are encoded by the ciliate's macronuclear genomes, including the genes for FeFe-hydrogenase, PFO and PNO. To achieve this, we

calculated CAI scores for each of the genes encoding these proteins, using the codon usage tables already generated for each species. For a given gene, a CAI score that falls within the upper 95% of that species' CAI distribution is consistent with the hypothesis that the gene was encoded by that genome.

To confirm that this was an effective method of distinguishing ciliate proteins from prokaryotes, for each ciliate CAI score distribution, we also calculated and then compared CAI score distributions for a species of Bacteria and an Archaea. For these analyses we selected the closest relatives of the most common bacterial and archaeal species in each transcriptome datasets, for which genome sequences were available in NCBI. CAI scores were calculated for each protein-coding gene of these species genomes, again using the codon usage tables inferred from each ciliate separately. The CAI score distributions for each ciliate, along with the CAI score distributions for the corresponding Bacteria and Archaea species, were then plotted as normalised distributions on the same axis so that they could be compared to one another directly. In each of the separate plots for *M. contortus*, *M. es*, *M. striatus*, *N. ovalis*, *C. porcatum*, *P. frontata* and *T. finlayi*, the ciliate CAI score distribution were clearly distinct from the Bacteria and Archaea CAI score distributions, suggesting that there were sufficient differences in codon usage bias to discriminate whether individual genes are from the ciliates or not.

For genes encoding either FeFe-hydrogenase, PFO and PNO from *M. contortus*, *M. es*, *M. striatus*, *N. ovalis*, *C. porcatum*, *P. frontata* and *T. finlayi*, the percentile rank of their CAI scores were determined, relative to the corresponding ciliate, Archaea and Bacteria CAI score distributions (Supp. Table 4.). For most genes, the percentile rank in the prokaryotic distributions was often 100% (12 of 21 cases), and always >90.5%, suggesting that, at least based on patterns of codon usage, these genes are unlikely to originate from prokaryote contaminants. By contrast, the same genes had codon usage profiles typical of bona fide ciliate genes in all cases (Table S4). The monophyly of these genes in all of the ciliates examined (Fig 4) provides additional tree-based evidence that is consistent with their origins from the ciliate macronuclear genomes. The CAI scores calculated for each of the proteins used to reconstruct the hydrogenosome metabolisms (Fig. 3) are also provided in Supplementary Data 1.

References for Supplementary Methods 1

RICE, P., LONGDEN, I. & BLEASBY, A. 2000. EMBOSS: the European molecular biology open software suite. *Trends in genetics,* 16**,** 276-277.

SHARP, P. M. & LI, W.-H. 1987. The codon adaptation index-a measure of directional synonymous codon usage bias, and its potential applications. *Nucleic acids research,* 15**,** 1281-1295.

Supplementary Table 1. The amount of (fastq) data generated for ciliates in the present study

	Genomic paired-end Illumina data (GB)	Transcriptomic paired-end Illumina data (GB)
Metopus contortus	42.565	61.192
Metopus striatus	34.872	3.938
Metopus es	61.972	3.406
Nyctotherus ovalis	14.400	11.993
Cyclidium porcatum	161.734	61.955
Plagiopyla frontata	22.573	55.232
Trimyema finlayi	23.655	4.420

Supplementary Table 2. Table of abbreviations used in Figure 3 (main manuscript)

**Abbreviation**	**Unabbreviated term**
ACO	Aconitase
ADP	Adenosine diphosphate
ALDO	Aldolase
AOX	Alternative oxidase
ASCT	Acetate:succinate CoA-transferase
ATP	Adenosine triphosphate
CI	Complex I
CII	Complex II
CIII	Complex III
CIV	Complex IV
CS	Citrate synthase
ENO	Enolase
ETC	Electron transport chain
FdO	Ferredoxin (oxidised)
FdR	Ferredoxin (reduced)
FH	Fumarate hydratase
GAPDH	Glyceraldehyde-3-Phosphate Dehydrogenase
GCK	Glucokinase
GCS	Glycine cleavage system
GCSH	Glycine cleavage system H protein
GCSL	Glycine cleavage system L protein
GCSP	Glycine cleavage system P protein
GCST	Glycine cleavage system T protein
GPI	Glucose-6-phosphate isomerase
HYD	FeFe-hydrogenase
IDH	Isocitrate dehydrogenase
ISC	Fe/S cluster biogenesis system
MDH	Malate dehydrogenase
MPP	Mitochondrial processing peptidase
mtDNA	Mitochondrial genome
NAD+	Nicotinamide adenine dinucleotide (oxidised)
NADH	Nicotinamide adenine dinucleotide (reduced)
NADP+	Nicotinamide adenine dinucleotide phosphate (oxidised)
NADPH	Nicotinamide adenine dinucleotide phosphate (reduced)
OGDH	Oxoglutarate dehydrogenase
OxAc	Oxaloacetate
PDH	Pyruvate dehydrogenase
PFK	Phosphofructokinase
PFO	Pyruvate:ferredoxin oxidoreductase
PGAM	Phosphoglycerate mutase
PGK	Phosphoglycerate kinase

P*i*	Inorganic phosphate
PK	Pyruvate kinase
PLP	Pyridoxal phosphate
PNO	Pyruvate:NADP+ oxidoreductase
SAM-e	S-adenosyl methionine
SCS	Succinyl coenzyme A synthetase
SDH	Succinate dehydrogenase
Suc	Succinate
Su-CoA	Succinyl coenzyme A
TCA cycle	Tricarboxylic acid cycle
TIM	Translocase of the inner membrane
TOM	Translocase of the outer membrane
TPI	Triosephosphate isomerase

Supplementary Table 3. Table showing MCF proteins detected from *Cyclidium porcatum*, *Metopus contortus* and *Plagiopyla frontata* and their closest homologues in *Saccharomyces cerevisiae*, inferred from phylogeny in Supplementary Figure 2.

**Name of MCF protein in *Saccharomyces***	**Transporter substrate**	**IDs of homologues from *Cyclidium porcatum***	**IDs of homologues from *Metopus contortus***	**IDs of homologues from *Plagiopyla frontata***
**AAC1/AAC2(PET9)/AAC3**	**ADP/ATP**	Cp23884<br>Cp18139<br>Cp15855	Mc30769	Pf14793
**SAL1**	**ADP/ATP**	Cp30842		
**YEA6/YIA6**	**NAD+**	Cp37172<br>Cp17843	Mc15577	
**PET8**	**S-adenosylmethionine (SAM-e)**		Mc23451	
**MME1**	**Magneisum**	Cp50430		
**MRS3/MRS4**	**Iron**	Cp15745	Mc27130	
**HEM25**	**Glycine**	Cp18467	Mc20557<br>Mc29888<br>Mc46598<br>Mc30514	Pf10386
**MTM1**	**Pyridoxal 5'-phosphate (PLP)**	Cp6146		
**PIC2**	**Copper/Phosphate**	Cp13858		
**YMC1/YMC2**	**Glycine**	Cp16277<br>Cp43145<br>Cp12094<br>Cp12093		
**OAC1**	**Oxaloacetate**	Cp16523		
	**Undetermined**	Cp1944<br>Cp13406<br>Cp15291<br>Cp17172<br>Cp17731<br>Cp18445<br>Cp18446<br>Cp18825<br>Cp30841<br>Cp40289	Mc2392<br>Mc4702<br>Mc13972<br>Mc17133<br>Mc18565<br>Mc18566<br>Mc18687<br>Mc21472<br>Mc21586<br>Mc24653<br>Mc25306<br>Mc25460<br>Mc25461<br>Mc26618<br>Mc27002<br>Mc27131<br>Mc28388<br>Mc28400<br>Mc28741<br>Mc29472<br>Mc29854<br>Mc30768<br>Mc31085<br>Mc31267<br>Mc32811<br>Mc48863<br>Mc50057<br>Mc50198<br>Mc68562	Pf1435<br>Pf2233<br>Pf9028<br>Pf11303<br>Pf13177<br>Pf13377<br>Pf13516<br>Pf14462<br>Pf38837
**ANT1 Peroxisomal transporter**	**Peroxisomal adenine transporter**			Pf10332<br>Pf10333<br>Pf9589

Supplementary Table 4. CAI scores for genes encoding FeFe-hydrogenase, PFO and PNO sequenced from ciliates in the present study and their corresponding percentile ranks relative to CAI score distributions for total genes from the same ciliate, an Archaea, and a Bacteria, as described in Supplementary Methods 1.

				Percentile rank of CAI scores within each distribution (Supp. Fig. 5)		
Species	Enzyme	ID	CAI score	Ciliate distribution	Archaea distribution	Bacteria distribution
Nyctotherus ovalis	FeFe-hydrogenase	No0001	0.825	85.1	100	100
	FeFe-hydrogenase	No0002	0.841	94.1	100	100
	FeFe-hydrogenase	No0003	0.826	85.9	100	100
Metopus contortus	FeFe-hydrogenase	Mc9793	0.785	54.7	98.6	96.9
	FeFe-hydrogenase	Mc10068	0.788	59.8	98.8	97.3
	FeFe-hydrogenase	Mc10161	0.785	54.7	98.6	96.9
Metopus es	FeFe-hydrogenase	Me0001	0.792	91.5	94.9	94.6
	FeFe-hydrogenase	Me0002	0.786	85.2	93.6	93.6
	FeFe-hydrogenase	Me0003	0.770	55.1	90.5	90.6
Metopus striatus	FeFe-hydrogenase	Ms0001	0.691	18.6	99.9	91.0
	FeFe-hydrogenase	Ms0002	0.727	55.7	100	95.8
	FeFe-hydrogenase	Ms0003	0.716	42.6	100	94.6
Plagiopyla frontata	FeFe-hydrogenase	Pf12774	0.679	31.3	99.7	99.4
	FeFe-hydrogenase	pf16078	0.702	47.4	99.1	98.4
Trimyema finlayi	FeFe-hydrogenase	Tf0001	0.697	43.3	100	100
Cyclidium porcatum	FeFe-hydrogenase	Cp400811	0.751	64.1	100	100
	FeFe-hydrogenase	Cp400821	0.781	86.5	100	100
	Pyruvate:NADP oxidoreductase	Cp433911	0.745	58.3	100	100
	Pyruvate:NADP oxidoreductase	Cp434321	0.780	58.9	100	100
	Pyruvate:NADP oxidoreductase	Cp182497	0.752	64.9	100	100
	Pyruvate:ferredoxin oxidoreductase	Cp182497	0.785	88.8	100	100

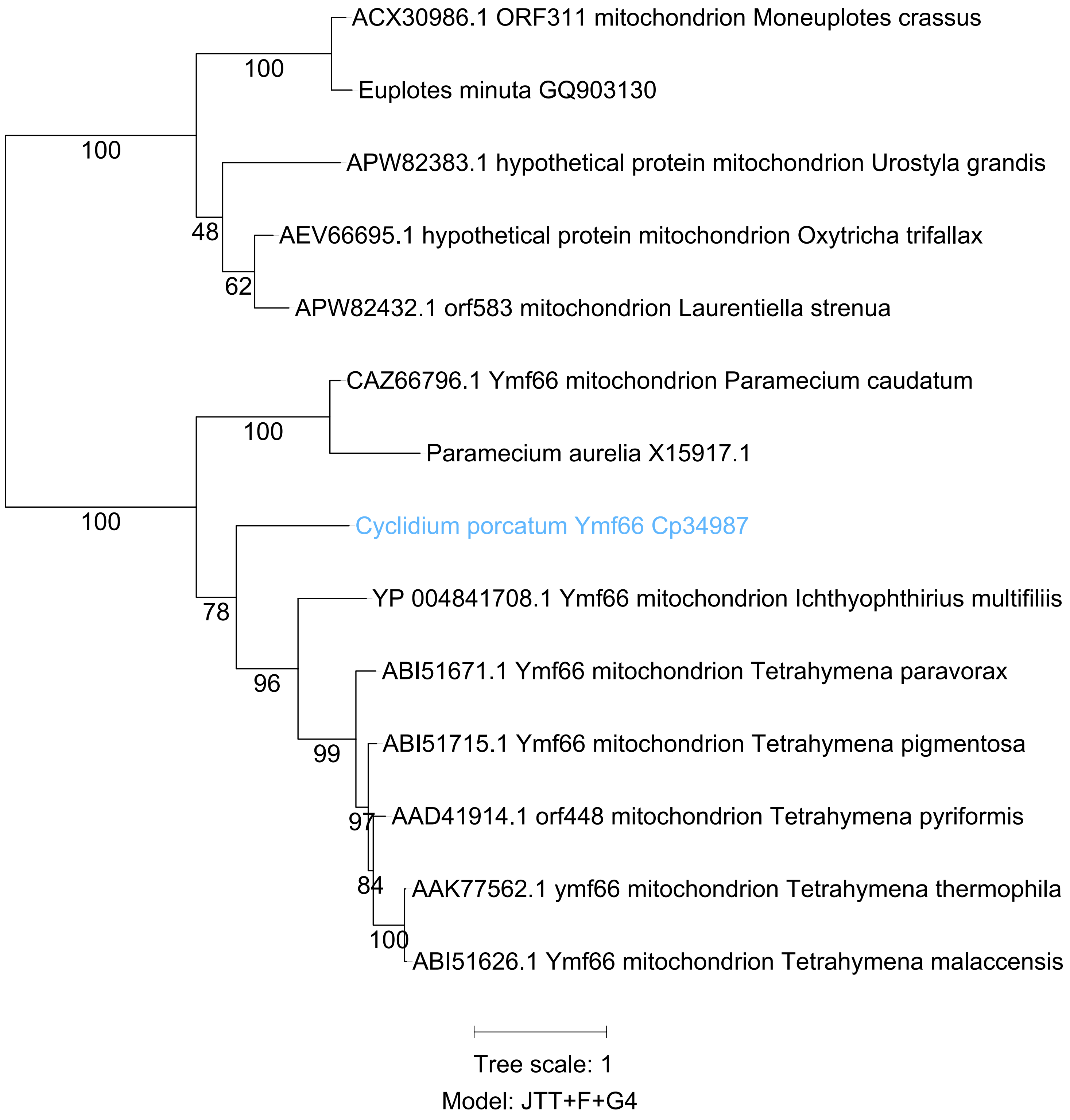

Supplementary Figure 1. Phylogeny of Ymf66 from *Cyclidium porcatum* and other ciliates. Tree inferred using the program IQ-TREE with 1000 ultrafast bootstrap.

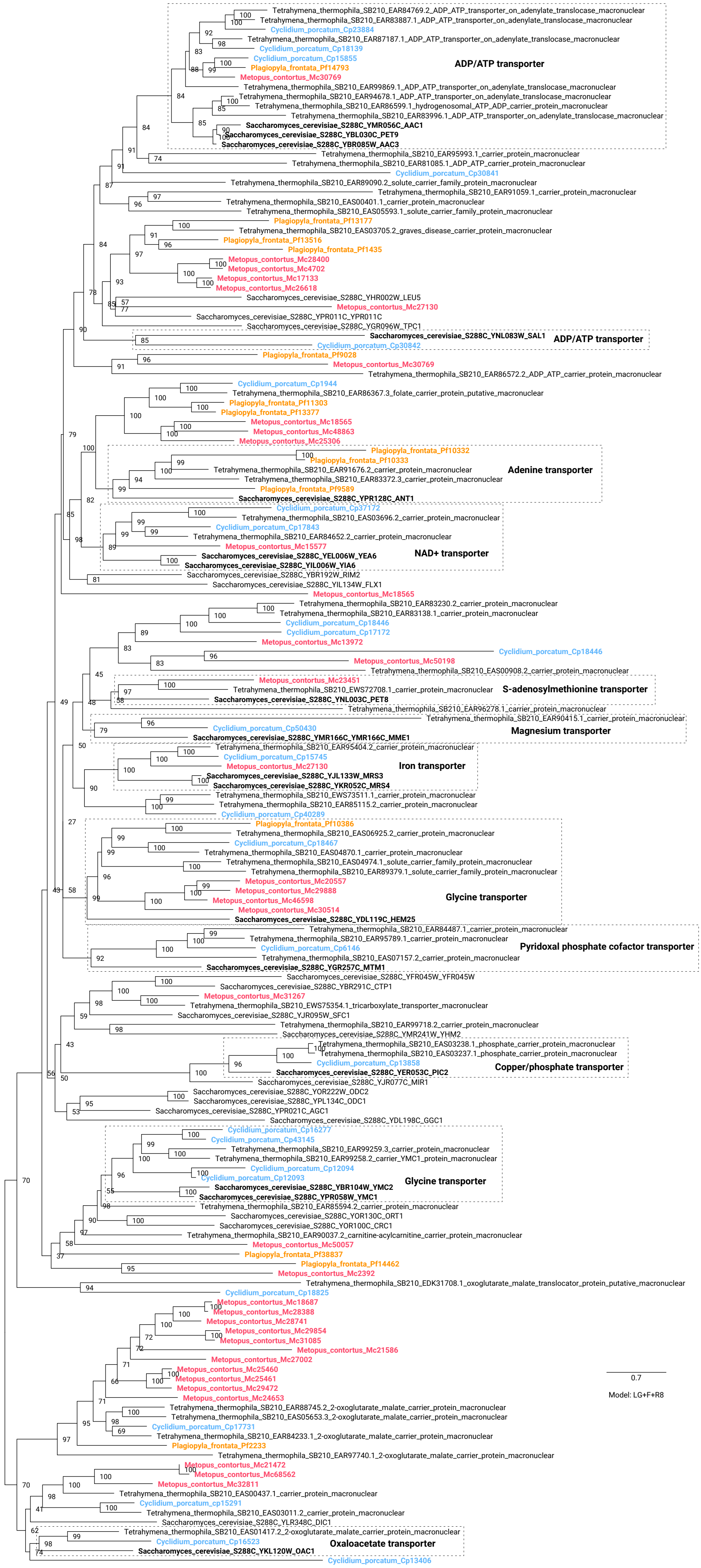

Tetrahymena_thermophila_SB210_EAR84769.2_ADP_ATP_transporter_on_adenylate_translocase_macronuclear
Tetrahymena_thermophila_SB210_EAR83887.1_ADP_ATP_transporter_on_adenylate_translocase_macronuclear
Cyclidium_porcatum_Cp23884
Tetrahymena_thermophila_SB210_EAR87187.1_ADP_ATP_transporter_on_adenylate_translocase_macronuclear
Cyclidium_porcatum_Cp18139
Cyclidium_porcatum_Cp15855
Plagiopyla_frontata_Pf14793
Metopus_contortus_Mc30769
ADP/ATP transporter
Tetrahymena_thermophila_SB210_EAR99869.1_ADP_ATP_transporter_on_adenylate_translocase_macronuclear
Tetrahymena_thermophila_SB210_EAR94678.1_ADP_ATP_transporter_on_adenylate_translocase_macronuclear
Tetrahymena_thermophila_SB210_EAR86599.1_hydrogenosomal_ATP_ADP_carrier_protein_macronuclear
Tetrahymena_thermophila_SB210_EAR83996.1_ADP_ATP_transporter_on_adenylate_translocase_macronuclear
Saccharomyces_cerevisiae_S288C_YMR056C_AAC1
Saccharomyces_cerevisiae_S288C_YBL030C_PET9
Saccharomyces_cerevisiae_S288C_YBR085W_AAC3
Tetrahymena_thermophila_SB210_EAR95993.1_carrier_protein_macronuclear
Tetrahymena_thermophila_SB210_EAR81085.1_ADP_ATP_carrier_protein_macronuclear
Cyclidium_porcatum_Cp30841
Tetrahymena_thermophila_SB210_EAR89090.2_solute_carrier_family_protein_macronuclear
Tetrahymena_thermophila_SB210_EAR91059.1_carrier_protein_macronuclear
Tetrahymena_thermophila_SB210_EAS00401.1_carrier_protein_macronuclear
Tetrahymena_thermophila_SB210_EAS05593.1_solute_carrier_family_protein_macronuclear
Plagiopyla_frontata_Pf13177
Tetrahymena_thermophila_SB210_EAS03705.2_graves_disease_carrier_protein_macronuclear
Plagiopyla_frontata_Pf13516
Plagiopyla_frontata_Pf1435
Metopus_contortus_Mc28400
Metopus_contortus_Mc4702
Metopus_contortus_Mc17133
Metopus_contortus_Mc26618
Saccharomyces_cerevisiae_S288C_YHR002W_LEU5
Metopus_contortus_Mc27130
Saccharomyces_cerevisiae_S288C_YPR011C_YPR011C
Saccharomyces_cerevisiae_S288C_YGR096W_TPC1
Saccharomyces_cerevisiae_S288C_YNL083W_SAL1
ADP/ATP transporter
Cyclidium_porcatum_Cp30842
Plagiopyla_frontata_Pf9028
Metopus_contortus_Mc30769
Tetrahymena_thermophila_SB210_EAR86572.2_ADP_ATP_carrier_protein_macronuclear
Cyclidium_porcatum_Cp1944
Tetrahymena_thermophila_SB210_EAR86367.3_folate_carrier_protein_putative_macronuclear
Plagiopyla_frontata_Pf11303
Plagiopyla_frontata_Pf13377
Metopus_contortus_Mc18565
Metopus_contortus_Mc48863
Metopus_contortus_Mc25306
Plagiopyla_frontata_Pf10332
Plagiopyla_frontata_Pf10333
Tetrahymena_thermophila_SB210_EAR91676.2_carrier_protein_macronuclear
Adenine transporter
Tetrahymena_thermophila_SB210_EAR83372.3_carrier_protein_macronuclear
Plagiopyla_frontata_Pf9589
Saccharomyces_cerevisiae_S288C_YPR128C_ANT1
Cyclidium_porcatum_Cp37172
Tetrahymena_thermophila_SB210_EAS03696.2_carrier_protein_macronuclear
Cyclidium_porcatum_Cp17843
Tetrahymena_thermophila_SB210_EAR84652.2_carrier_protein_macronuclear
Metopus_contortus_Mc15577
Saccharomyces_cerevisiae_S288C_YEL006W_YEA6
NAD+ transporter
Saccharomyces_cerevisiae_S288C_YIL006W_YIA6
Saccharomyces_cerevisiae_S288C_YBR192W_RIM2
Saccharomyces_cerevisiae_S288C_YIL134W_FLX1
Metopus_contortus_Mc18565
Tetrahymena_thermophila_SB210_EAR83230.2_carrier_protein_macronuclear
Tetrahymena_thermophila_SB210_EAR83138.1_carrier_protein_macronuclear
Cyclidium_porcatum_Cp18446
Cyclidium_porcatum_Cp17172
Metopus_contortus_Mc13972
Cyclidium_porcatum_Cp18446
Metopus_contortus_Mc50198
Tetrahymena_thermophila_SB210_EAS00908.2_carrier_protein_macronuclear
Metopus_contortus_Mc23451
Tetrahymena_thermophila_SB210_EWS72708.1_carrier_protein_macronuclear
S-adenosylmethionine transporter
Saccharomyces_cerevisiae_S288C_YNL003C_PET8
Tetrahymena_thermophila_SB210_EAR96278.1_carrier_protein_macronuclear
Tetrahymena_thermophila_SB210_EAR90415.1_carrier_protein_macronuclear
Cyclidium_porcatum_Cp50430
Magnesium transporter
Saccharomyces_cerevisiae_S288C_YMR166C_YMR166C_MME1
Tetrahymena_thermophila_SB210_EAR95404.2_carrier_protein_macronuclear
Cyclidium_porcatum_Cp15745
Metopus_contortus_Mc27130
Iron transporter
Saccharomyces_cerevisiae_S288C_YJL133W_MRS3
Saccharomyces_cerevisiae_S288C_YKR052C_MRS4
Tetrahymena_thermophila_SB210_EWS73511.1_carrier_protein_macronuclear
Tetrahymena_thermophila_SB210_EAR85115.2_carrier_protein_macronuclear
Cyclidium_porcatum_Cp40289
Plagiopyla_frontata_Pf10386
Tetrahymena_thermophila_SB210_EAS06925.2_carrier_protein_macronuclear
Cyclidium_porcatum_Cp18467
Tetrahymena_thermophila_SB210_EAS04870.1_carrier_protein_macronuclear
Tetrahymena_thermophila_SB210_EAS04974.1_solute_carrier_family_protein_macronuclear
Tetrahymena_thermophila_SB210_EAR89379.1_solute_carrier_family_protein_macronuclear
Metopus_contortus_Mc20557
Metopus_contortus_Mc29888
Glycine transporter
Metopus_contortus_Mc46598
Metopus_contortus_Mc30514
Saccharomyces_cerevisiae_S288C_YDL119C_HEM25
Tetrahymena_thermophila_SB210_EAR84487.1_carrier_protein_macronuclear
Tetrahymena_thermophila_SB210_EAR95789.1_carrier_protein_macronuclear
Cyclidium_porcatum_Cp6146
Pyridoxal phosphate cofactor transporter
Tetrahymena_thermophila_SB210_EAS07157.2_carrier_protein_macronuclear
Saccharomyces_cerevisiae_S288C_YGR257C_MTM1
Saccharomyces_cerevisiae_S288C_YFR045W_YFR045W
Saccharomyces_cerevisiae_S288C_YBR291C_CTP1
Metopus_contortus_Mc31267
Tetrahymena_thermophila_SB210_EWS75354.1_tricarboxylate_transporter_macronuclear
Saccharomyces_cerevisiae_S288C_YJR095W_SFC1
Tetrahymena_thermophila_SB210_EAR99718.2_carrier_protein_macronuclear
Saccharomyces_cerevisiae_S288C_YMR241W_YHM2
Tetrahymena_thermophila_SB210_EAS03238.1_phosphate_carrier_protein_macronuclear
Tetrahymena_thermophila_SB210_EAS03237.1_phosphate_carrier_protein_macronuclear
Cyclidium_porcatum_Cp13858
Copper/phosphate transporter
Saccharomyces_cerevisiae_S288C_YER053C_PIC2
Saccharomyces_cerevisiae_S288C_YJR077C_MIR1
Saccharomyces_cerevisiae_S288C_YOR222W_ODC2
Saccharomyces_cerevisiae_S288C_YPL134C_ODC1
Saccharomyces_cerevisiae_S288C_YPR021C_AGC1
Saccharomyces_cerevisiae_S288C_YDL198C_GGC1
Cyclidium_porcatum_Cp16277
Cyclidium_porcatum_Cp43145
Tetrahymena_thermophila_SB210_EAR99259.3_carrier_protein_macronuclear
Tetrahymena_thermophila_SB210_EAR99258.2_carrier_YMC1_protein_macronuclear
Cyclidium_porcatum_Cp12094
Cyclidium_porcatum_Cp12093
Glycine transporter
Saccharomyces_cerevisiae_S288C_YBR104W_YMC2
Saccharomyces_cerevisiae_S288C_YPR058W_YMC1
Tetrahymena_thermophila_SB210_EAR85594.2_carrier_protein_macronuclear
Saccharomyces_cerevisiae_S288C_YOR130C_ORT1
Saccharomyces_cerevisiae_S288C_YOR100C_CRC1
Tetrahymena_thermophila_SB210_EAR90037.2_carnitine-acylcarnitine_carrier_protein_macronuclear
Metopus_contortus_Mc50057
Plagiopyla_frontata_Pf38837
Plagiopyla_frontata_Pf14462
Metopus_contortus_Mc2392
Tetrahymena_thermophila_SB210_EDK31708.1_oxoglutarate_malate_translocator_protein_putative_macronuclear
Cyclidium_porcatum_Cp18825
Metopus_contortus_Mc18687
Metopus_contortus_Mc28388
Metopus_contortus_Mc28741
Metopus_contortus_Mc29854
Metopus_contortus_Mc31085
Metopus_contortus_Mc21586
Metopus_contortus_Mc27002
Metopus_contortus_Mc25460
Metopus_contortus_Mc25461
Metopus_contortus_Mc29472
Metopus_contortus_Mc24653
Tetrahymena_thermophila_SB210_EAR88745.2_2-oxoglutarate_malate_carrier_protein_macronuclear
Tetrahymena_thermophila_SB210_EAS05653.3_2-oxoglutarate_malate_carrier_protein_macronuclear
Cyclidium_porcatum_Cp17731
Tetrahymena_thermophila_SB210_EAR84233.1_2-oxoglutarate_malate_carrier_protein_macronuclear
Plagiopyla_frontata_Pf2233
Tetrahymena_thermophila_SB210_EAR97740.1_2-oxoglutarate_malate_carrier_protein_macronuclear
Metopus_contortus_Mc21472
Metopus_contortus_Mc68562
Metopus_contortus_Mc32811
Tetrahymena_thermophila_SB210_EAS00437.1_carrier_protein_macronuclear
Cyclidium_porcatum_cp15291
Tetrahymena_thermophila_SB210_EAS03011.2_carrier_protein_macronuclear
Saccharomyces_cerevisiae_S288C_YLR348C_DIC1
Tetrahymena_thermophila_SB210_EAS01417.2_2-oxoglutarate_malate_carrier_protein_macronuclear
Cyclidium_porcatum_Cp16523
Oxaloacetate transporter
Saccharomyces_cerevisiae_S288C_YKL120W_OAC1
Cyclidium_porcatum_Cp13406
0.7
Model: LG+F+R8

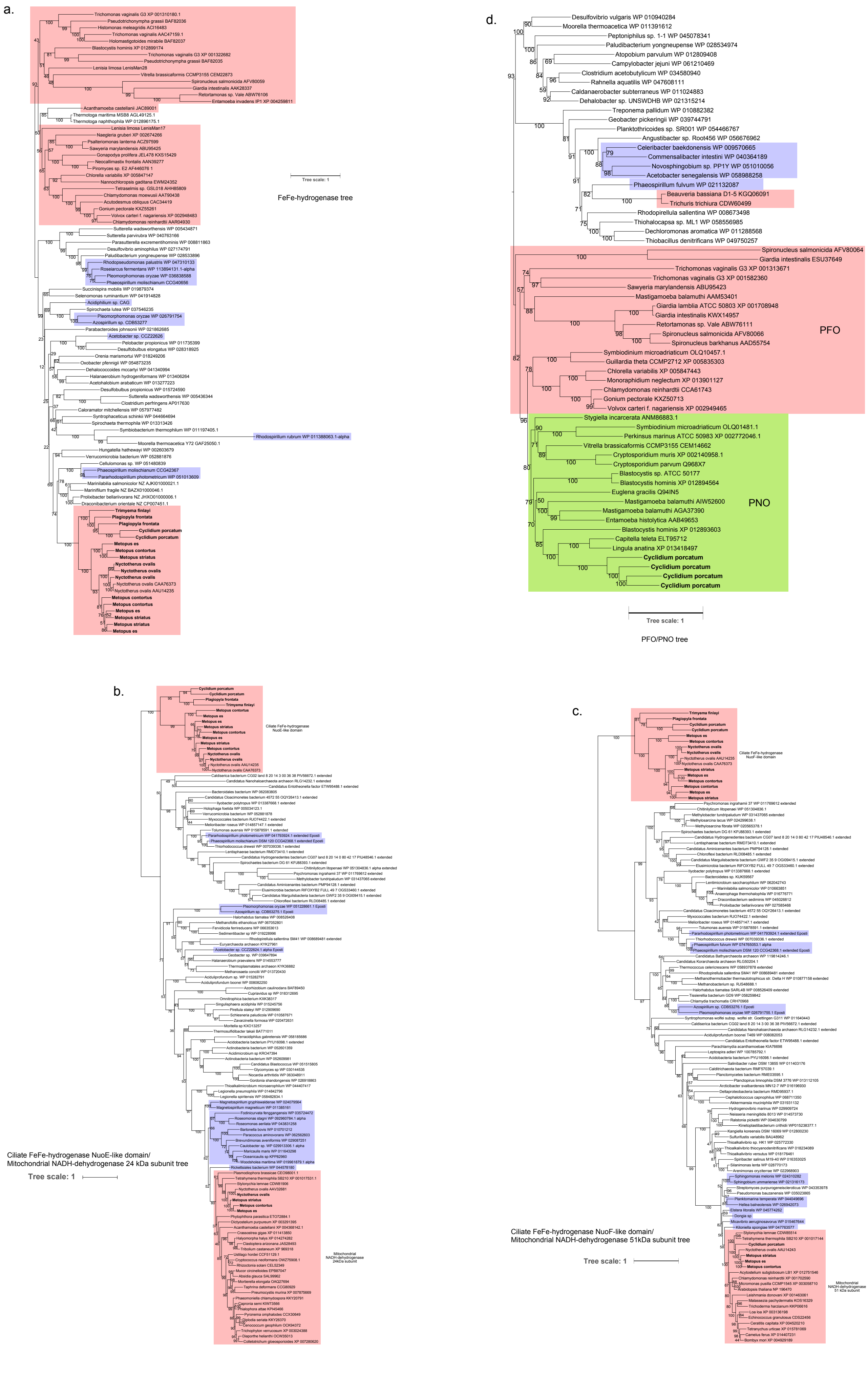

Supplementary Figure 3, a. Phylogeny of FeFe-hydrogenase; b. Phylogeny of mitochondrial 24 kDa subunit of NADH-dehydrogenase, prokaryotic NuoE and the NuoE-like domain of ciliate FeFe-hydrogenase; c. Phylogeny of mitochondrial 51 kDa subunit of NADH-dehydrogenase, prokaryotic NuoF and the NuoF-like domain of ciliate FeFe-hydrogenase; d. Phylogeny of PFO and PNO. These trees are the same trees shown in Figure 4, d. (main manuscript) but here all of the branches are displayed, with no clades collapsed.

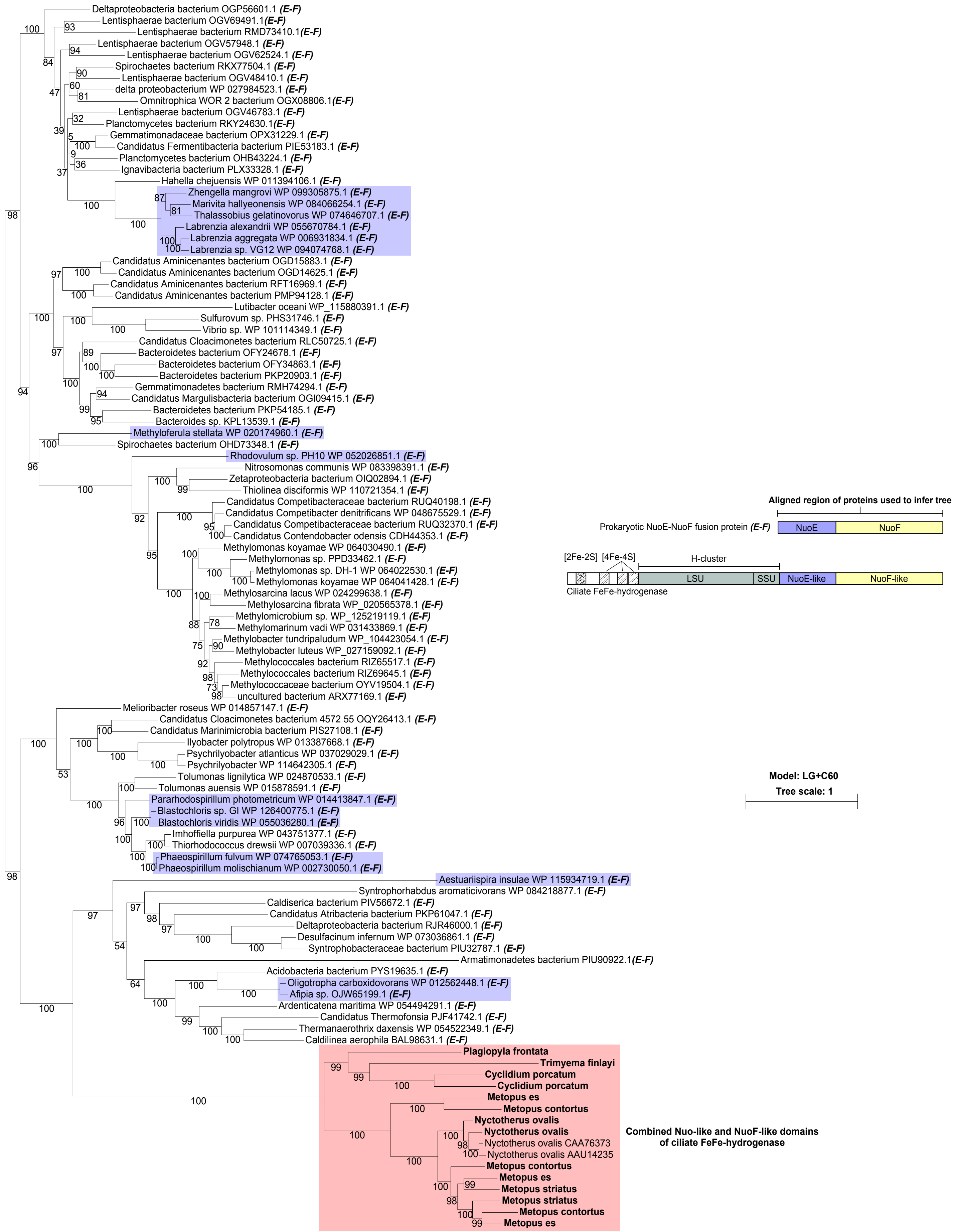

Supplementary Figure 4. A phylogeny inferred from an alignment of prokaryotic NuoE-NuoF fusion proteins and the NuoE-like and NuoF-like C-terminal domains of ciliate FeFe-hydrogenases. Sequences from the present study are in bold text, prokaryotic NuoE-NuoF fusion protein sequences are labelled ***(E-F)***, sequences highlighted in red are from eukaryotes (which in this case only includes sequences from ciliates) and sequences from alphaproteobacteria are highlighted in blue.

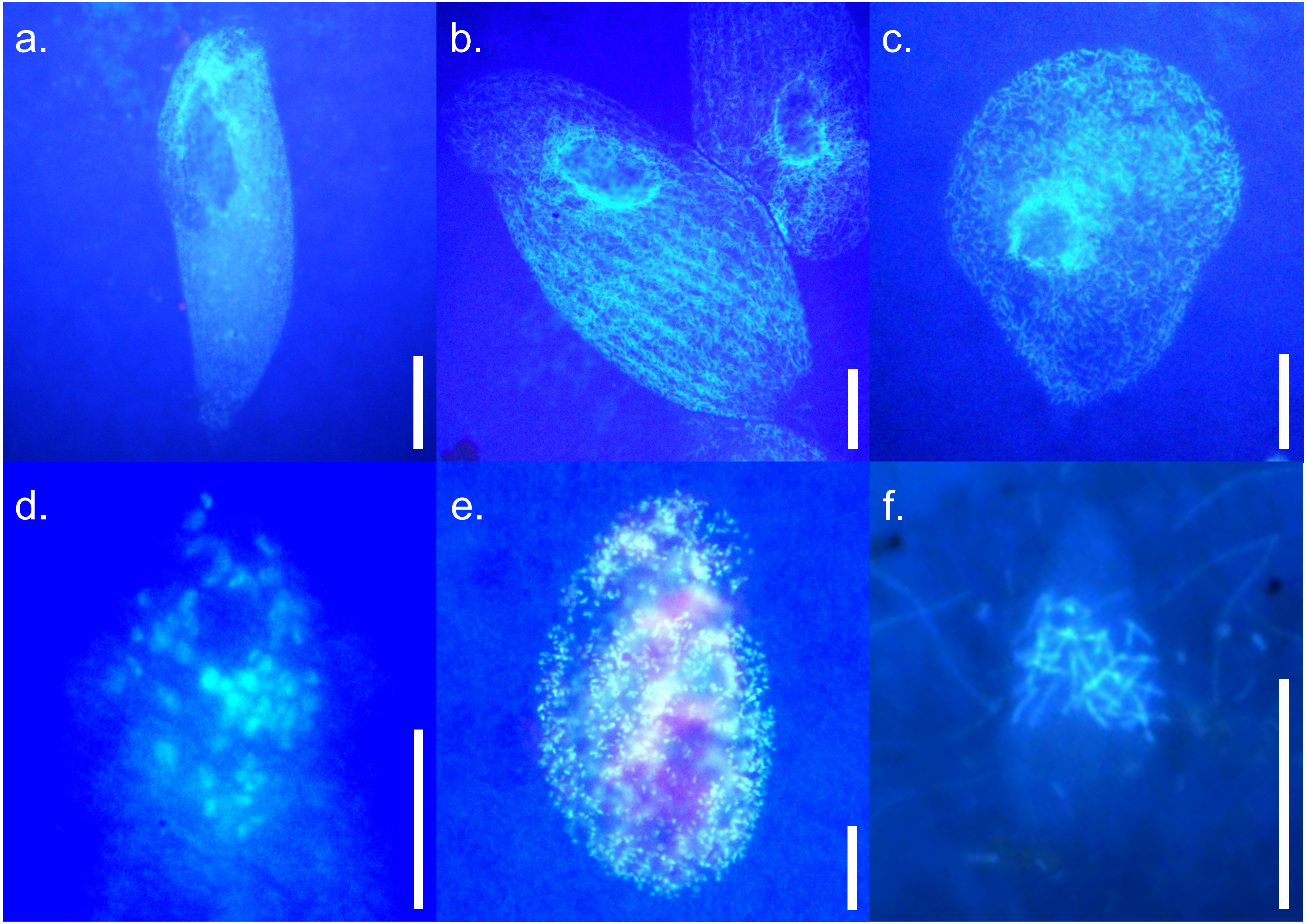

Supplementary Figure 5. F420 autofluorescence of endosymbiotic methanogens imaged *in situ* from the ciliates (a.) *Metopus contortus*, (b.) *Metopus es*, (c.) *Metopus striatus*, (d.) *Trimyema finlayi*, (e.) *Plagiopyla frontata* and (f.) *Cyclidium porcatum*. Scale bars: 20μm

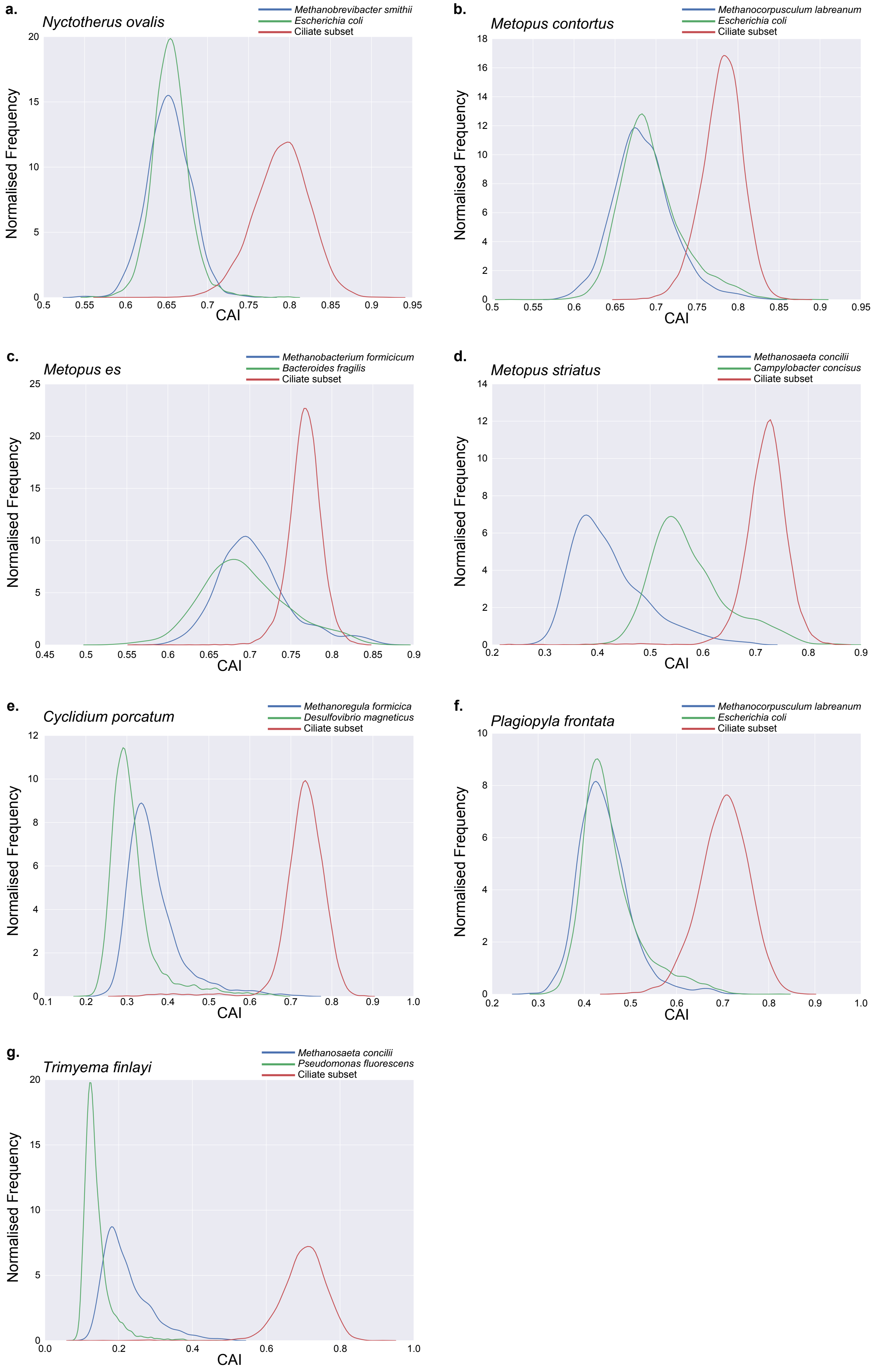

Supplementary Figure 6. Normalised frequency distribution plots of codon adaptation index (CAI) scores for genes from (a.) *Nyctotherus ovalis*, (b.) *Metopus contortus*, (c.) *Metopus es*, (d.) *Metopus striatus*, (e.) *Cyclidium porcatum*, (f.) *Plagiopyla frontata*, (g.) *Trimyema finlayi* (Described in Supplementary Methods 1).
